# Driving anger dimensions and their relationship with aberrant driver behavior in Lebanon: Results from a national self-reported survey

**DOI:** 10.1371/journal.pone.0283293

**Published:** 2023-03-17

**Authors:** Dalal Youssef, Pascale Salameh, Linda Abou-Abbas, Louis-Rachid Salmi

**Affiliations:** 1 ISPED School of Public Health, Bordeaux University, UMR_S 1219—Research Center Bordeaux Population Health (BPH), Bordeaux, France; 2 Clinical Trial Program, Ministry of Public Health, Beirut, Lebanon; 3 Lebanese Higher Institute of Technical & Professional (IPNET), Beirut, Lebanon; 4 Institut National de Santé Publique, Epidémiologie Clinique et Toxicologie (INSPECT-LB), Beirut, Lebanon; 5 School of Medicine, Lebanese American University, Byblos, Lebanon; 6 Department of Research, Faculty of Pharmacy, Lebanese University, Hadat, Lebanon; 7 Department of Primary Care and Population Health, University of Nicosia Medical School, Nicosia, Cyprus; 8 Neuroscience Research Center, Faculty of Medical Sciences, Lebanese University, Beirut, Lebanon; Sunway University, MALAYSIA

## Abstract

Driving anger may vary across countries due to culture. This might affect driver behavior, which, in turn, impacts the driving outcomes. This study aims to investigate the relationship between socio-demographic variables, driving anger, and the self-reported aberrant behavior among Lebanese drivers and to determine which anger dimension is linked to driving behavior. A cross-sectional study was conducted among eligible Lebanese drivers from all Lebanese governorates. Data were collected using an anonymous Arabic self-reported questionnaire that included demographic information, driving-related variables, and two scales: the Driver Behavior Questionnaire (DBQ) and the Driver Anger Scale (DAS). Four hierarchical regressions were performed taking the DBQ subscales as the dependent variable and the DAS subscales as independent variables. Out of 1102 surveyed drivers, 68.4% were males, having a mean age of 34.6 ± 12.3 years and an average driving experience of 13.5 ± 10.8 years. DBQ, DAS, and their subscales showed good reliability. Older age and female gender were negatively associated with the tendency of committing aggressive violations. However, being a professional driver and increasing annual mileage were positively associated with a higher tendency to commit aggressive violations. In addition to these factors, a higher educational level was found associated with a lower risk of driver’s involvement in traffic violations. However, increased driving experience was associated with a higher tendency to commit aggressive violations. Reported driving errors were also found positively associated with older age, increasing mileage, and being a professional driver. However, larger driving experience and higher education were found protectors from erroneous behavior. Hierarchical regression showed that anger prompted by hostile gesture, discourtesy, police presence, traffic obstruction, and slow driving were positively associated with aggressive violations. All the DAS subscales were found to be positively associated with ordinary violations. traffic obstruction was also found associated with a higher tendency of drivers to commit lapses, as well as anger, which originated from police presence and slow driving which were found also positively associated with errors. Driver anger dimensions were found positively associated with aberrant driver behavior. To overcome road anger, there is a need to train drivers on coping strategies to restrain aberrant driving behavior.

## Introduction

The increase in motorization and mobility has brought attention to road safety and the role of driver behavior in road traffic crashes (RTCs). Many studies have focused on exploring the human factors affecting driving behavior that can affect global patterns of traffic and pose risks to other road users [1–3]. Several methods have been used for gathering and analyzing data on driver behavior, including surveys, naturalistic driving studies, simulation studies, and event data recorders. Of note, surveys are the most common method for gathering information on a wide range of driver behaviors. However, these methods could be used alone or in combination to gain a comprehensive view of driver behavior and help researchers to understand the underlying causes of accidents and to develop interventions to improve safety.

The Driver Behavior Questionnaire (DBQ) is a widely used instrument to examine drivers’ self-reported aberrant behaviors that increase the risk of RTCs [4]. The original version of the DBQ, developed by Reason et al. (1990), consists of 50 items classified into three dimensions: violations, errors, and lapses [5]. Violations are distinct from errors and lapses as they cover behaviors that deliberately contravene safe driving practices, while errors are defined as a failure of a planned sequence of mental or physical activities in the selection of an action to achieve the intended goal [6]. Lapses are defined as an unwitting deviation of action from intention [5]. The DBQ instrument evaluates the driver behavior under a theoretical framework and reflects the metacognitive skills of the drivers.

Driving anger, which refers to the emotional state of displeasure, fury, or rage experienced by drivers, has also been a focus in the driving safety literature. It is considered a special case of cognitive distraction and has been linked to personality traits and common road situations such as heavy traffic, impeding events, and the behavior of other road users [7]. Similar to cognitive distraction, driving anger can withdraw attention from the information necessary for safe operation of a vehicle, leading to a mental impairment. Studies have found anger to be one of the most important factors involved in unsafe driving [8, 9]. The Driving Anger Scale (DAS) is widely used to measure the propensity of drivers to become angry while encountering potentially anger-provoking driving situations [7]. It can measure six types of driving anger: hostile gestures, slow driving, traffic obstruction, illegal driving, and police presence anger. Anger can interfere with cognitive processes such as attention and judgement, leading to excessive confidence and lower risk perception among angered drivers [10, 11]. It is unclear whether anger leads to RTCs due to increased cognitive load and unintentional failures or due to deliberate unsafe driving behaviors.

A close relationship has been found between anger and driving behavior, but it is complex and not always positive [12, 13]. Studies have found that drivers with higher levels of anger are more likely to commit violations on the road [9, 14–16]. However, the relationship between anger and errors is mixed, with some studies finding a positive association and others finding no significant association [16–18]. Some researchers have found different associations between different types of driving anger and driving aberrations [13]. These findings highlight the importance of investigating the relationship between anger and aberration on a subscale level. Furthermore, the association between socio-demographic variables and aberrant driving behaviors is an important aspect to consider when investigating the factors that contribute to road traffic crashes. The type of driver, such as professional versus non-professional, as well as factors like age, gender, and driving experience, can all have a significant impact on aberrant driving behaviors and should be considered in studies exploring this topic [19–22].

In Lebanon, road traffic injuries are the leading cause of unintentional injury and death with an estimated fatality rate of 18.1 per 100,000 population [23]. According to the statistics issued by the Lebanese Ministry of Interior, 2666 RTCs were recorded in 2020 leading to 337 fatalities [24]. Of note, driver behavior was one of the main reported causes of the RTCs in the country. In addition, the transportation system is facing several problems such as bad road infrastructure, a lack of public transportation, an increasing number of vehicles, traffic congestion, and RTCs. In addition, it is well known that traffic congestion in urban areas can lead to discomfort and road rage among drivers. Since driving anger was found in the literature very likely to be associated with aberrant driving behaviors leading to RTCs among drivers [25], therefore, it is of great interest to examine the effects of driving anger on driving behavior among Lebanese drivers. This is reasonable driver behavior explains a significant amount of variance in traffic accidents than do vehicle, roadway, or environmental factors and so far, studies on road user behaviors in Lebanon are scarce and no previous research in Lebanon has investigated such kind of relationship. However, it is important to note that the Driving Anger Scale (DAS) and Driver Behavior Questionnaire (DBQ) subscales have different psychological characteristics and using overall scores from these scales may mask the real associations at the subscale level [25]. Therefore, for a more accurate understanding of the relationship between driving anger and aberrant driving, DAS and DBQ subscales will be used in this study, rather than overall scores. This will allow for a consideration of both the effect of anger on unintentional cognitive failure "errors" and deliberate infringements of accepted principles of safe driving "violations". The findings of this study are expected also to contribute to driver behavior research by providing a more detailed understanding of how anger and aberrant driving behaviors are related. Such in-depth exploration is needed to help researchers and policymakers understand which interventions are more effective and to determine the best approach to apply practical driving safety education. In addition, investigating the association between driving anger and sociodemographic characteristics has strong substantial impacts on driver selection and driver education which in turn could improve road safety in the country.

The present study aims to investigate, based on self-reported data, the relationship between socio-demographic variables, driving anger, and the self-reported aberrant behavior among Lebanese drivers and to determine which anger dimension is linked to driving behavior.

## Materials and methods

### Participants and sampling

A cross-sectional study was conducted using a convenience sampling method among Lebanese drivers aged 18 years and above from all Lebanese governorates over the period extending from November to December 2019. The research protocol was properly evaluated and approved, ensuring adequate protection of study participants and no risk was imposed on them. Eligible participants were active drivers who were 18 years old or older, had a valid driver’s license, drove regularly, and agreed to participate. Drivers who were not currently practicing driving activities or rarely drove (less than once per month) were excluded, as well as illiterate drivers who were unable to understand the questions.

### Questionnaire

An anonymous, Arabic, self-completion questionnaire was used for data collection. The questionnaire includes a brief introduction to the background, the objective of the study, and instructions for filling out the questionnaire. None of the survey questions asked for information that could harm the participant in any way.

The questionnaire was composed of three main sections: The first part of the questionnaire consisted of socio-demographic variables, including gender, age, professional or non-professional driving status, and other driver-related information such as driving experience, and annual mileage, obtained from participants. The driving experience was defined as “years of active driving” instead of “years licensed” because drivers may be licensed but inactive (rarely drive after obtaining a driving license) and some Lebanese drivers begin to drive before obtaining a driving license.

The second part of the questionnaire included the following scales:

### Driving anger scale (DAS)

Driving anger was measured using the Arabic version of original of the Driving Anger Scale (DAS) validated among Lebanese drivers [26]. This Arabic version of the instrument contains 30 anger-provoking scenarios and respondents were requested to imagine each situation happening to them and then to rate the amount of anger experienced for each situation on a five-point Likert scale (1 = not angry, 2 = slightly angry, 3 = angry, 4 = very angry 5 = extremely angry). These are examples of scenarios described in the instrument designed to provoke anger in the respondents: “someone in front of you does not start up when the light turns green” and “a pedestrian walks slowly across the middle of the street slowing you”… Higher scores reflect more anger. The Arabic version of DAS has shown good internal consistency (α  =  0.93) and showed that a six-factor structure of DAS (hostile gesture, traffic obstruction, discourtesy, illegal driving, slow driving, and police presence) best fits the Lebanese drivers’ data. The α-values of these subscales ranged between 0.81 and 0.92, indicating an excellent internal consistency [26].

### Driver behavior questionnaire (DBQ)

The 45-item Lebanese version of the Driver Behavior Questionnaire (DBQ) was utilized to measure the main forms of self-reported aberrant driving behaviors [27]. It was adapted from the original 50-item version of DBQ [5]. Each item describes a particular aberrant driving behavior. Participants are instructed to consider their driving behavior and to indicate how often each aberration occurred to them in the past 12 months on a six-point scale (1 = never; 2 = hardly ever; 3 = occasionally; 4 = quite often; 5 = frequently; 6 = nearly all the time) across different driver situations. Items scores are summed, with higher scores indicating more frequent aberrant behavior. The adapted Lebanese version of DBQ demonstrated that the four factors of DBQ measured four types of aberrant behaviors: aggressive violation, ordinary violations, errors, and lapses. The four subscales were moderately correlated, and all had good internal consistency [27].

### Data collection procedure

Face to face approach was used to collect data from eligible participants after getting their acceptance to participate. Potential respondents were recruited from public places (shopping malls, parking stations…) and universities by two trained data collectors who were students in the traffic major at the Lebanese Higher Technical School. Data collectors were responsible to explain orally the study objectives for the individual subjects before their participation and providing them with general instructions needed for the study completion. Participants were reassured that their participation in the study is free and entirely voluntary. Written informed consent was obtained from each participant as well. No financial compensation was given to respondents for their participation. Data were gathered anonymously and handled confidentially. The questionnaire took 10 minutes to be filled.

### Statistical analysis

The collected data was entered and analyzed using the statistical software SPSS (Statistical Package for Social Sciences), version 24.0. The person who performed data entry was not involved in the data collection process.

Since missing data constituted < 10% of the total database, it was not substituted. The distribution of items of DBQ and those of DAS scales was checked for normality. The normal distribution of these variables was established by visual examination of the histogram, with skewness and kurtosis lower than 1 [28]. Descriptive analyses were done using counts and percentages for categorical variables and mean and standard deviation for continuous measures. A bivariate analysis was conducted using the ANOVA test to compare the means of the DBQ subscales and the categorical variables. Pearson correlation was used for linear correlation between continuous variables. All variables that showed a *p*-value< 0.2 in the bivariate analysis were included in the model as independent variables to assess the correlates of dependent variables (DBQ subscales) in the whole sample. To statistically “control” sociodemographic variables and to see whether adding DAS subscales significantly improves the model’s ability to predict the driver behavior, four hierarchical regressions, which are a special type of linear regression, were performed taking the DBQ subscales as the dependent variable and the DAS subscales as independent variables to explore how the aberrant driving behaviors could be predicted by a combination of variables.

In each analysis, variables are added to the model in separate steps called “blocks. This aimed to unravel the sole contribution of each set of variables in explaining the aberrant driving behaviors. Therefore, the demographic variables were entered in the first step.

Sociodemographic variables (age, gender, annual mileage, driving experience, and occupation) were introduced into the model in the first step as controlled variables. The six subscales of DAS were entered in the second step when performing the hierarchical regression. Of note, to provide linear regression, the indicators had to be modified and the number of categories was reduced (categories were merged in cases where there was no significant difference). The statistical significance level was set at 0.05.

## Results and discussion

### Baseline information

Out of the 1550 eligible subjects recruited for the study, 1200 (77.4%) individuals were willing to participate. A total of 1,102 (91.8%) questionnaires were collected. The age of the participants ranged from 18 to 82, with a mean age of 34.6 ± 12.3 years. Most participants were male (68.4%), had an education level higher than secondary (57.6%), and were working in a field other than professional driving (57.2%). The mean driving experience was 13.5 ± 10.8 years. Most participants (77.3%) owned their own vehicle, which was mostly a two-wheel motor vehicle (71.1%). More than 30% of respondents traveled more than 10,000 kilometers annually. 43.2% of surveyed drivers were involved in road traffic collisions and 46.8% had been penalized for traffic offenses in the previous 3 years.

### DBQ and DAS subscales

For DBQ subscales, the α-values ranged between 0.68 and 0.814, indicating good internal consistency (Table 1). The overall reliability of the DBQ-L scale was good (α = 0.85). Of the four subscales, the highest mean score was for aggressive violations followed by ordinary violations, lapses, and errors. With regards to DAS, the α-values obtained from subscales ranged between 0.87 and 0.93, indicating an excellent internal consistency. The overall reliability of the DAS-A scale was good (α = 0.93).

**Table 1:** Summary statistics for the DBQ and DAS subscales among Lebanese drivers.

	#	Number of items	Item mean	Scale means	S.D	α	Skewness	Kurtosis
	**DBQ subscales**							
F1	Aggressive violations	**5**	2.996	14.99	2.05	0.68	0.075	0.654
F2	Ordinary violations	**10**	2.656	26.56	4.72	0.79	0.213	0.634
F3	Errors	**15**	2.218	33.27	5.92	0.814	0.967	0.589
F4	Lapses	**15**	2.52	37.80	4.80	0.73	0.579	0.521
	DBQ scale	45	2.51	113.14	14.55	0.85	0.336	0.714
	**DAS subscales**							
D1	Discourtesy	**9**	3.34	30.09	7.51	0.91	-0.21	-0.94
D2	Traffic obstruction	**7**	3.39	23.91	5.15	0.90	0.72	0.97
D3	Slow driving	**6**	3.01	18.29	5.54	0.92	0.18	-0.80
D4	Police presence	**4**	2.33	9.37	4.16	0.91	0.24	-0.98
D5	Illegal driving	4	3.62	14.53	2.28	0.87	0.27	-0.67
D6	Hostile gesture	**3**	3.79	11.38	1.91	0.92	0.27	-0.67
	**DAS scale**	**33**	3.24	107.54	16.78	0.93	0.37	-0.3

**N.B:** S.D refers to standard deviation, alpha referred to Cronbach alpha, min for minimum, and max for maximum, Vehicle/Env refers to the vehicle and environment dimension.

### Correlation between driver anger and driver behavior (DBQ-L) subscales

Aggressive violations were found to be positively correlated to hostile gesture (r = 0.140), slow driving (r = 0.184), discourtesy (r = 0.071), police presence (r = 0.163) and traffic obstruction (r = 0.092).Similarly, ordinary violations subscale was positively correlated to discourtesy (r = 0.101), police presence (r = 0.215), traffic obstruction (r = 0.061), slow driving(r = 0.177) and illegal driving (r = 0.112). Meanwhile, lapses were only positively correlated to police presence (r = 0.216) and traffic obstruction (r = 0.114). Errors were positively correlated to anger prompted by the police presence(r = 0.284) and slow driving (r = 0.111). Interestingly, only one correlation was found between the anger instigated by illegal driving and the violation subscale (Table 2).

**Table 2:** Pearson correlations between DAS and DBQ-L subscales.

	Hostile gesture	Illegal driving	Discourtesy	Police presence	Traffic obstruction	Slow driving
**Aggressive violation**	0.040*	0.030	.071*	.163**	.092**	.184**
**Ordinary violation**	0.007	0.112*	.101**	.215**	.061*	.177**
**Lapses**	0.012	0.015	0.025	.216**	.114**	.130**
**Errors**	-0.029	0.014	0.007	.284**	0.052	.111**

Note: Pearson correlations ** p < 0.01. * p < 0.05., CI: Confidence interval 95%.

### DBQ-L and socio-demographic variables

Gender differences revealed that men reported a higher tendency to commit aggressive violations (p = 0.048), and ordinary violations (p = 0.049), compared to females (Table 3). The four dimensions of aberrant driver behavior (aggressive violations, ordinary violations, errors, and lapses) were significantly affected by the occupation and the educational level of the surveyed drivers. Drivers with lower levels of education (secondary education or less) were more likely to be involved in aberrant driving behaviors, such as aggressive violations, ordinary violations, errors, and lapses. Ordinary violation (r = -0.559, p < 0.001) and lapses (r = -0.544, p < 0.001), were negatively correlated with age. Younger drivers were more likely to be involved in aberrant driving behaviors, such as ordinary violations, and lapses. However, errors (r = 0.442, p < 0.001) were positively associated with age and had a different pattern where errors decreased for young drivers.

**Table 3:** DBQ-L and sociodemographic characteristics of the Lebanese drivers.

	Sociodemographic variables	N	Mean	S.D	F-value	P-value	Cohen’s d
** **	**Gender**						
**Aggressive violations**	Men	754	15.03	2.11	1.132	0.048	0.069
	Women	348	14.89	1.90			
**Ordinary violations**	Men	754	26.81	4.69	1.275	0.049	0.073
	Women	348	26.46	4.80			
**Lapses**	Men	754	37.84	4.97	0.112	0.738	0.022
	Women	348	37.73	4.44			
**Errors**	Men	754	33.23	5.85	0.097	0.755	0.02
	Women	348	33.35	6.08			
** **	**Occupation**						Cohen’s f
**Aggressive violations**	Student	272	15.21	2.02	9.950	<0.001	0.182
	Driver	116	15.79	2.25			
	Worker but not as a driver	630	14.76	2.01			
	Not working	84	14.87	1.77			
**Ordinary violations**	Student	272	27.65	5.11	36.510	<0.001	0.338
	Driver	116	29.84	6.20			
	Worker but not as a driver	630	25.55	3.81			
	Not working	84 26.17		4.54			
**Lapses**	Student	272	38.32	5.18	10.090	<0.001	0.167
	Driver	116	39.67	7.01			
	Worker but not as a driver	630	37.23	4.14			
	Not working	84	37.86	3.60			
**Errors**	Student	272	34.52	6.24	42.990	<0.001	0.338
	Driver	116	37.76	7.05			
	Worker but not as a driver	630	31.84	4.86			
	Not working	84	33.65	6.40			
** **	**Education Level**						Cohen’d
**Aggressive violations**	Secondary or less	468	2.18	0.10	3.200	0.07	0.109
	University or above	634	1.94	0.08			
**Ordinary violations**	Secondary or less	468	5.08	0.23	10.111	<0.001	0.194
	University or above	634	4.41	0.17			
**Lapses**	Secondary or less	468	5.12	0.24	8.540	<0.001	0.178
	University or above	634	4.53	0.18			
**Errors**	Secondary or less	468	6.49	0.30	15.06	<0.001	0.237
	University or above	634	5.39	0.21			
** **	**Annual mileage**						Cohen’ f
**Aggressive violation**	0–2000 km	136	14.69	1.71	5.164	<0.001	0.119
	2000–4000 km	154	14.56	1.68			
	4000–6000 km	234	14.87	2.11			
	6000–10000 km	242	15.39	2.06			
	> 10000 km	336	15.09	2.20			
	**Annual mileage**				9.228	<0.001	0.19
**Ordinary violation**	0–2000 km	136	25.73	4.32			
	2000–4000 km	154	25.20	3.36			
	4000–6000 km	234	26.12	4.22			
	6000–10000 km	242	27.66	4.72			
	> 10000 km	336	27.05	5.46			
	**Annual mileage**				3.054	0.016	0.098
**Lapses**	0–2000 km	136	37.48	4.70			
	2000–4000 km	154	37.03	3.83			
	4000–6000 km	234	37.35	3.84			
	6000–10000 km	242	38.30	4.82			
	> 10000 km	336	38.25	5.70			
	**Annual mileage**				7.665	<0.001	0.174
**Errors**	0–2000 km	136	32.84	5.09			
	2000–4000 km	154	31.61	5.07			
	4000–6000 km	234	32.68	5.39			
	6000–10000 km	242	34.69	6.27			
	> 10000 km	336	33.58	6.43			
** **	**Age**				**Pearson**	**P-value**	CI 95%
**Aggressive violation**		1102	34.64	12.33	-0.047	0.043	(-0.231-(-0.032)
**Ordinary violation**					-.559**	<0.001	(-0.712; -0.412)
**Lapses**					-.544**	<0.001	(-0.609; -0.354)
**Errors**					.442**	<0.001	(0.312–0.567)
	**Driving experience**						
**Aggressive violation**		1102	13.46	10.76	-0.038	0.203	(-0.212; -0.019)
**Ordinary violation**					-.142**	<0.001	(-0.287; -0.089)
**Lapses**					-.135**	<0.001	(-0.361; -0.281)
**Errors**					.161**	<0.001	(0.069;0.312)

Additionally, aggressive violations (p<0.001), ordinary violations (p<0.001), errors (p<0.001) and lapses (p = 0.016<0.001) increased with annual mileage. Despite the decrease of violations (r = -0.142, p<0.001) and lapses (r = -0.135, p<0.001) with increasing driving experience, errors were found increasing with driving experience (r = 0.161, p<0.001).

### Hierarchical regression

As a result of the hierarchical regression exploring the factors associated with aggressive violations, the first model that included in its first step of regression analysis the sociodemographic factors, explains 16% of the variation and was statistically significant (p<0.001) (Table 4). Older age ((β = -0.084, 95% CI (-0.127: -0.009), p = 0.018) and female gender ((β = -0.181, 95% CI (-0.353: -0.089) p = 0.048) were protector factors against committing aggressive violations. However, being a professional driver ((β = 0.126, 95% CI (0.029–0.527), p = 0.001) and increasing annual mileage ((β = 0.058, 95% CI (0.033–0.174), p = 0.045) were positively associated with the involvement of driver in aggressive violations. In the second step, the six subscales of DAS were included in the model in addition to the demographic variables which additionally explain 11% of the model and it is also statistically significant. Anger prompted by hostile gesture (β = 0.125, 95% CI(0.030–0.169), p = 0.047), discourtesy (β = 0.015, 95% CI(0.009–0.190), p = 0.014), police presence (β = 0.114, 95% CI (0.103–0.352), p<0.001), traffic obstruction (β = 0.152, 95% CI(0.137–0.312), p = 0.014), and slow driving (β = 0.142, 95% CI(0.109–0.484), p<0.001), were found positively associated with aggressive violation. In terms of ordinary violation, the first model explains 9.8% of the variation (p<0.001). Being female, increasing age, and higher educational level were found protective factors reducing the risk of drivers’ involvement in traffic violations. On the contrary, extensive years of driving experience, increased annual mileage, and being a professional driver were associated with violations among drivers. The second step explains 15.4% of the variation with 5.1% further explanation than demographic variables. All the DAS subscales instigated by hostile gestures(β = 0.145, 95% CI (0.037–0.325), p = 0.04), discourtesy(β = 0.099, 95% CI (0.076–1.176), p = 0.009), traffic obstruction(β = 0.116, 95% CI (0.078–0.194), p<0.001, police presence(β = 0.151, 95% CI (0.034–0.578), p<0.001), illegal(β = 0.075, 95% CI (0.036–0.097), p = 0.039) and slow driving(β = 0.114, 95% CI (0.042–0.431), p<0.001 were found to be associated with of ordinary violations. The variation of errors was explained with 11.3% by demographic variables. Larger driving experience and education higher than secondary level were protectors from erroneous driving behavior. Conversely, older age, increasing annual mileage, and working as a professional driver were positively associated with the errors among drivers. The inclusion of DAS subscales increased the explanation of errors by 6.2%. Anger originated from police presence (β = 0.227, 95% CI (0.130–0.685), p<0.001) and slow driving (β = 0.164, 95% CI (0.101–0.972), p = 0.045) were positively associated with erroneous driving behavior. However, no association was found between the remaining DAS subscales such as hostile gestures, traffic obstruction, discourtesy, illegal driving, and the errors committed while driving.

**Table 4:** Summary of hierarchical multiple regression results of DAS associations with aberrant driver behavior.

Model		Unstandardized coefficients β	Standardized coefficients β	t	P	95% C.I		Model summary		
						**Lower**	**Upper**	**R** ^**2**^	**Δ R** ^**2**^	**Sig**
**Aggressive violations**	Age	-0.932	-0.084	-1.565	**0.018**	-0.127	-0.009	0.16	0.16	<0.001
	Gender	-0.751	-0.181	-0.257	**0.048**	-0.353	-0.089			
	Year of experience	-0.198	-0.006	-0.1	0.921	-0.025	0.016			
	Occupation	2.138	0.126	3.902	**0.001**	0.029	0.527			
	Education Level	-0.659	-0.029	-0.909	0.363	-0.426	0.101			
	Annual mileage	1.312	0.058	1.785	**0.045**	0.033	0.974			
	Hostile gesture	2.984	0.125	0.323	**0.047**	0.030	0.169	0.27	0.11	<0.001
	Illegal driving	23.11	0.025	0.663	0.508	0.041	0.194			
	Discourtesy	4.302	0.015	0.367	**0.014**	0.009	0.190			
	Police presence	0.929	0.114	3.725	**<0.001**	0.103	0.352			
	Traffic obstruction	1.319	0.152	1.627	**0.014**	0.137	0.312			
	Slow driving	2.121	0.142	4.157	**<0.001**	0.109	0.484			
**Ordinary violations**	Age	-0.513	-0.054	-1.052	**0.023**	-0.087	-0.043	0.098	0.098	<0.001
	Gender	1.412	0.064	2.126	**0.034**	-0.165	0.933			
	Year of experience	0.918	0.144	2.721	**0.007**	0.167	1.057			
	Occupation	2.315	0.196	6.382	**<0.001**	0.129	0.238			
	Education Level	-0.789	-0.066	-2.191	**0.029**	-1.902	-0.040			
	Annual mileage	2.731	0.107	3.407	**0.001**	0.024	0.644			
	Hostile gesture	4.231	0.145	1.478	**0.04**	0.037	0.725	0.154	0.051	<0.001
	Illegal driving	1.632	0.075	2.062	**0.039**	0.036	0.097			
	Discourtesy	0.789	0.099	2.629	**0.009**	0.076	1.176			
	Police presence	5.213	0.151	5.175	**<0.001**	0.034	0.878			
	Traffic obstruction	0.598	0.116	0.529	**0.017**	0.078	0.194			
	Slow driving	0.319	0.114	3.498	**<0.001**	0.042	0.431			
**Errors**	Age	0.312	0.02	0.395	**0.013**	0.014	0.084	0.113	0.113	<0.001
	Gender	0.716	0.048	1.609	0.108	0.015	0.976			
	Year of experience	1.873	0.205	3.92	**<0.001**	0.021	0.489			
	Occupation	2.121	0.216	7.134	**<0.001**	0.193	1.123			
	Education Level	-0.592	-0.076	-2.582	**0.01**	-1.027	-0.026			
	Annual mileage	1.981	0.176	2.453	**0.014**	0.779	1.542			
	Hostile gesture	0.431	0.056	1.862	0.063	0.036	1.063	0.175	0.062	<0.001
	Illegal driving	0.332	0.047	1.312	0.19	0.032	1.238			
	Discourtesy	0.159	0.063	1.69	0.091	-0.016	0.106			
	Police presence	2.312	0.227	7.913	**<0.001**	0.130	0.685			
	Traffic obstruction	1.419	0.032	1.049	0.294	-0.300	0.261			
	Slow driving	3.315	0.164	2.011	**0.045**	0.101	0.972			
**Lapses**	Age	0.122	0.053	0.991	0.317	0.008	0.074	0.056	0.056	<0.001
	Gender	-0.501	-0.013	-0.109	0.913	-0.711	-0.007			
	Year of experience	-0.206	-0.219	-4.02	**0.001**	-0.353	-0.058			
	Occupation	1.512	0.078	2.456	**0.014**	0.577	1.724			
	Education Level	-0.497	-0.081	-2.637	**0.008**	-0.494	-0.022			
	Annual mileage	0.708	0.088	2.718	**0.007**	0.020	0.970			
	Hostile gesture	-0.431	-0.027	-0.853	0.394	-0.658	0.261	0.094	0.047	<0.001
	Illegal driving	-0.313	-0.004	-0.106	0.916	-0.601	0.631			
	Discourtesy	-0.193	-0.036	-0.92	0.358	-0.723	-0.026			
	Police presence	1.012	0.167	5.561	**<0.001**	0.119	1.068			
	Traffic obstruction	0.687	0.086	2.723	**0.007**	0.046	0.651			
	Slow driving	0.719	0.087	2.589	**0.01**	0.045	0.823			

**Note:** The reference group for gender categorical variable is male drivers; the reference group for educational level is secondary or less; for annual mileage, the reference group was ≤ 6000km; for occupation, the reference group was the "nonprofessional driver". Weighed model.

Regarding Lapses, the first model including only socio-demographic variables explained 5.6% of the variation. Alike the errors dimension, larger driving experience and education higher than secondary level were negatively associated with lapses and increasing annual mileage and working as a driver were negatively associated with erroneous driving behavior. Conversely, they were associated with errors among drivers. However, the second step which comprised DAS dimensions described additionally 4.7% of the lapses. Only anger provoked by the presence of police (β = 0.167, 95% CI (0.119–1.068), p<0.001), traffic obstruction (β = 0.086, 95% CI (0.046–0.651), p = 0.007) and the slow driving (β = 0.087, 95% CI (0.045–0.823), p = 0.01) were found positively associated with lapses.

## Discussion

Driving behavior is a complex and multidisciplinary research domain and aberrant driving behaviors that threaten the safety of road users should be avoided. This could not be achievable without unveiling the factors that reside behind such behavior. This study conducted on Lebanese driver sample revealed that several factors, including age, gender, occupation, education, annual mileage, and driving experience, were associated with aberrant driving behaviors, specifically aggressive and ordinary violations. Younger age, male gender, being a professional driver, and lower educational level were found to be positively associated with a higher tendency to commit these types of violations. Additionally, the study found that errors were positively associated with older age, increasing mileage, and working as a driver, while larger driving experience and higher education were negatively associated with erroneous behavior. Furthermore, the study identified specific dimensions of driving anger (hostility, discourtesy, police presence, traffic obstruction, and slows driving) that were associated with aggressive violations. All the DAS subscales were found associated with ordinary violations and some DAS subscales were found to be positively correlated with errors and lapses. Overall, the study highlights the need for understanding the complex factors that contribute to aberrant driving behaviors in order to develop effective interventions to improve road safety.

In terms of socio-demographic variables, gender, age, education level, and occupation were found to be associated with aberrant driving behaviors. Specifically, the study found that males had a higher tendency to commit aggressive and ordinary violations, while no significant difference in self-reported driving errors was found across gender. This could be related to the fact that men have a higher tendency to force others to get out of the way and consider it a submissive action. Similar results were found in previous studies where it was also stated that males exhibited driving violations more frequently than females [5, 29–32]. Our results in terms of errors are consistent with the findings of the study conducted by Reason et al. [5]. Of note, other studies have reported a higher tendency in females to report lapses and errors which was not found among Lebanese drivers [5, 30, 32, 33]. Although the stereotype is that men are more focused on one task while driving and women are more cautious and able to multitask, this study found that women were more likely to commit errors and shortcomings related to space perception while driving. This finding may be due to Lebanese women’s desire to conceal their weaknesses while driving in order to avoid stereotypes. Another possible explanation is that women tend to be more cautious while driving, which may lead to fewer errors.

As for age, our study revealed a decreased pattern of aggressive and ordinary violations with increased age. Younger age was found to be positively correlated with an increase in both violations scores (ordinary and aggressive). This could be related to the increased maturity with age, which might mellow drivers and make them less likely to adopt aggressive driving behavior. Consistently with other studies, our findings showed that men and younger drivers are more likely to display aggressive violations while driving. Additionally, older age was found to be positively associated with self-reported errors. This could be related to the decline in cognitive and physical abilities that often comes with age. It highlights the importance of interventions and programs aimed at older drivers to help improve their driving skills and safety on the road. Overall, this study provides important insights into the socio-demographic factors associated with aberrant driving behaviors and the need to address these factors in order to improve road safety for all road users [34].

The study found that being a professional driver was positively associated with a higher tendency to commit aggressive and ordinary violations. One possible explanation is that professional drivers often have higher workloads and tight time schedules, which may lead to increased stress levels and a greater likelihood of engaging in risky driving behaviors [35]. In other words, they were more exposed to higher traffic volumes on the road and to a situation-specific emotional state that could affect their behavior. This was consistent with the results of a study conducted in Qatar that reported a higher likelihood of risky driving among professional drivers [36]. The study also highlighted the importance of addressing this issue, as professional drivers play a significant role in the traffic system of Lebanon, and there is a need to improve the ’Traffic Safety Culture’ among Lebanese professional drivers. Additionally, it is important to examine and understand the personal acceptance of risky driving behaviors among professional drivers in order to develop effective interventions to improve road safety [37].

Additionally, a higher educational level (university degree or above) was found to be a protective factor from committing ordinary violations, lapses, and errors while driving. In other words, education could provide the driver with the necessary knowledge and skills to navigate the road safely and make better decisions while driving. Furthermore, the study highlights the importance of investing in educational programs and awareness campaigns targeting younger, less educated drivers and professional drivers to improve road safety in Lebanon.

In terms of driving-related variables, our findings showed that all dimensions of risky behavior (errors, lapses, aggressive and ordinary violations were positively associated with the increased annual mileage, whereas violations increased with larger driving experience. Comparable results were reported by a study conducted among Australian drivers [6]. However, beginner drivers lack confidence in their driving performance and are still in the phase of confidence-building. Hence, the limited driving experience in handling various traffic situations makes them more anxious to perpetrate a traffic violation [38]. When they get more experience, they will feel more familiar with the traffic environment and increase, therefore, their involvement in traffic violations. Similarly, a study concerning the traffic safety culture of professional drivers in Qatar indicated that experienced professional drivers are more likely to perform risky driving behaviors [36].

As for the driving errors dimension, it was also found affected by driver characteristics. This was consistent with other studies [18, 39, 40]. More specifically, age, education as well as driving experience were found to have the highest effect on driving error in our present research. Remarkably, errors in our study exhibited a different pattern where it was found that errors increased with age indicating that older drivers are more likely to make driving errors. This could be explained that young drivers have better mental and physical characteristics than older drivers reducing their likelihood of committing errors. In comparison with our findings, a previous study has found that errors decrease for young drivers but remained quite unchangeable with age within the older samples [6]. Both drivers’ experience and education were protective factors from erroneous behavior indicating that a more experienced and more educated driver is less likely to perform driving errors. This finding probably means that both of these characteristics help drivers to properly manage a potentially dangerous situation and protect themselves from committing an error. It is noteworthy that there is confusion and disparity in the literature in terms of age and experience and their impact on aberrant driver behavior. A specific insight was provided by Wells and al. [41], who reported that older drivers had consistently lower violation scores than younger drivers. In this case, the effects of age on errors could be explained by the fact that novice drivers are still in the learning phase, hence improving their skills and reducing their driving errors. A driving simulator study about errors and violations during training of pre-licensed drivers showed similar results [42]. At a later age, acquired driving skills can be weakened and a further increase in errors could be expected.

Our study also showed that aberrant driver behavior subscales were positively correlated to anger subscales such as hostile gestures, illegal driving, slow driving, discourtesy, police presence, and traffic obstruction. Different anger triggers were found to have differing impacts on aberrant driving behaviors in our study. This result is consistent with the finding of a study conducted by Zhang et al. [13]. Similar to other studies, our findings suggested that the relationship between driving anger and driver behavior depends on different natures of anger or situational characteristics, respectively [13, 43–45].

Based on the frustration-aggression hypothesis, aggressive driving is a result of anger or frustration caused by thwarted goals [46]. Our study also found that anger prompted by hostile gestures; discourtesy, police presence; traffic obstruction, and slow driving were positively associated with aggressive violations. This finding is consistent with the driving anger factors reported by Zhang and Chan’s study [13], which either block the goal to drive safely, the ‘‘general survival goal”, or the arrival goal. Previous studies showed that hindrance anger trigger was associated with aggressive driving [13, 44]. In addition, the relationship between anger and aggressive expressions has been reported in numerous studies [17, 47]. Hence, driving anger was positively associated with aberrant driving behaviors in the sense of deliberate infringements that are most aggressive and targeted at other road users. Angry drivers attempt to influence other drivers, either to achieve their driving goals or to take revenge for other road users’ driving behaviors. These results are alarming especially because the anger–aggression correlation might have become stronger in more recent years, given the increasing traffic congestion problem worldwide.

For ordinary violations, all the DAS subscales instigated by hostile gestures, discourtesy, traffic obstruction, and police presence, illegal and slow driving were found positively correlated with ordinary violations [17, 47]. Many studies highlighted such kind of correlation between anger and violations. This highlights the importance of all aspects of anger originating from any driving situation described in the 33 items of DAS in leading to traffic violations and also stressed the importance to mitigate factors triggering anger on road.

In this study, we found that only anger originated from police presence and slow driving was positively associated with erroneous driving behavior. Apart from triggering aggressive violations and deliberate traffic infractions, anger can negatively impact driving behavior, since research has demonstrated that anger could compromise the cognitive performance of drivers including judgment and reasoning which may rise the possibility of making errors [48]. Of note, the effects of driving anger on errors have been explored also in simulation-based studies where Jeon et al. found that angry drivers made more errors than the control group [49], and Stephens et al. showed that drivers who had exhibited a higher level of anger in a simulated driving situation were also more projected to miss dangerous traffic hazards [50]. Furthermore, a recent meta-analysis found a significant correlation of 0.179 between driving anger and errors [12]. However, previous investigations have also reported weak or non-significant relationships between driving anger and errors [51].

With regards to lapses, our findings revealed that anger provoked by the presence of police, traffic obstruction, or slow driving could lead to lapses while driving. These results were consistent with the findings of a study conducted about dimensions of driving anger and their relationships with aberrant driving among Chinese drivers which demonstrated that the three dimensions of driving anger have different relations with aberrant driving behaviors [13].

### Strengths and limitations

To the best of the authors’ knowledge, this is the first Lebanese study to explore how driving anger affects driver behavior and to determine which anger dimension is linked to aberrant driving behavior. This investigation thus offers special insight into the interaction between driving anger, driving behavior, and other road users. Hence, our findings guide researchers and policymakers about the interventions that are more effective for Lebanese drivers in order to reduce the number of accidents and victims of aberrant driving.

Several limitations should be acknowledged in our study. First, the study’s cross-sectional design in nature does not allow deriving causal relationships of the main associations and has no dimension of time. Despite weighing over gender, age, and geographical regions, selection bias might be present, as the sample included a low percentage of older drivers aged above 60 years. The low percentage of older participants recruited here is not surprising since older people were not sufficiently interested in participating in such kind of study compared to young people. Hence, there is concern regarding the generalizability of the findings to the older driver’s population. Future research exploring the validity of the identified relations between anger–aberration for older drivers is recommended. Besides, our data rely on self-reported measures, the data from which may suffer from social desirability bias. Hence, aberrant driving behaviors and driver anger may be underreported due to the deliberate propensity of participants to give responses in a manner that will be noticed favorably by others. However, the anonymity of survey might have offset the negative impact of social desirability. Some researchers have also found that self-reported driving aberrations were only slightly affected by social desirability bias since the aberrant driving behavior assessed with DBQ is strongly associated with the observed driver behavior on the road [52]. Further work is required to validate the findings suggested in this study using naturalistic driving data.

### Implications

This study provided insights about the relationships between driving anger and driving behavior, which can be useful for researchers and policymakers in addressing this issue. The findings that young and male drivers were more involved in aggressive and ordinary violations suggest that targeted treatment programs and intervention strategies may be necessary to reduce driving anger among these groups. This could include measures such as encouraging defensive driving and promoting a positive perspective among drivers. Additionally, the research highlights the need for further study to be directed towards specific driver groups, such as professional drivers, in order to gain a more complete understanding of the factors that contribute to driving anger and aberrant driving behavior. In light of the fact that the actions of other drivers cannot always be controlled, it is important for each driver to take responsibility for their own behavior on the road. One way to do this is to encourage drivers to not take the actions of other drivers personally. To this end, it is recommended that educational training on defensive driving be offered as a practical way to deal with frustration and anger on the road, in addition to anger management programs to improve drivers’ skills. Additionally, enforcing laws related to avoiding driver violations and distractions is essential to reduce the risk of aberrant driving behavior. The study also highlights the need for further research to examine the extent to which driving anger and driver behavior are associated with an increased risk of road traffic crashes.

## Conclusion

This study found that driving anger and its dimensions were positively associated with aggressive and ordinary violations, as well as driving errors. The results suggest that anger can distract drivers from processing important information while driving, leading to deliberate and aggressive behaviors and an increase in driving errors. However, the impact of driving anger was found to depend on the triggers of anger. To address this issue and reduce the likelihood of aberrant driving behavior, it is important to provide drivers with training in coping strategies to manage their anger while driving. This could include techniques such as deep breathing, visualization, and cognitive reframing, as well as strategies for avoiding or managing anger triggers.

## Declarations

### Ethics approval and consent to participate

As research involving the use of survey procedures are exempted from ethical approval in Lebanon [53] and the risks of this research are no more than minimal, this study was exempted from ethical approval by the Ministry of Public Health. All methods were performed in accordance with the ethical standards as laid down in the Declaration of Helsinki and its later amendments. The study design assured adequate protection of study participants, and neither included clinical data about patients nor configured itself as a clinical trial. Written informed consent was obtained for each participant. They were reassured that their participation is voluntary and that they were free to withdraw at any time. In addition, all information was gathered anonymously and handled confidentially.

## Supporting information

S1 Checklist (DOCX)

S1 File (DOCX)
