# Supplementary material for: Driving anger dimensions and their relationship with aberrant driver behavior in Lebanon: Results from a national self-reported survey
Source: PLoS One. 2023 Mar 17;18(3):e0283293. doi: 10.1371/journal.pone.0283293 (PMC10022756; doi:10.1371/journal.pone.0283293)
Supplement: S1 File — (DOCX) [file pone.0283293.s002.docx]

# Anger hits the road: Validation and cross-cultural adaptation of the Arabic version of driving anger scale and its association with road traffic crashes and fines

Dalal Youssef^1,2,3^, Pascale Salameh^4,5,6,7^, Linda Abou-Abbas^8^, Louis-Rachid Salmi^1^

**Corresponding author:**

Dalal Youssef, MSc, MPH, RSM, Ph.D., Bordeaux Research Center for Population Health, Institut de santé publique, d'épidémiologie et de développement (ISPED), Bordeaux University, France & Clinical trial Program, Ministry of Public Health, Beirut, Lebanon & Lebanese Higher Institute of Technical & Professional, Bir Hassan, Ministry of Education, Beirut, Lebanon ORCID IDs: [https://orcid.org/0000-0003-3085-6849](https://orcid.org/0000-0003-3085-6849?lang=en) Email: [dalal.youssef@u-bordeaux.fr](mailto:dalal.youssef@u-bordeaux.fr) ; [dyoussef@moph.gov.lb](mailto:dyoussef@moph.gov.lb); [dalalyoussef.esu@gmail.com](mailto:dalalyoussef.esu@gmail.com); Phone number: +96171239633

1. ISPED school of public Health, Bordeaux University, France, UMR_S 1219 - Research Center Bordeaux Population Health (BPH), Bordeaux, France
2. Clinical trial Program, Ministry of Public Health, Beirut, Lebanon
3. Lebanese Higher Institute of Technical & Professional (IPNET), Beirut, Lebanon
4. School of Medicine, Lebanese American University, Byblos, Lebanon
5. Department of Research, Faculty of Pharmacy, Lebanese University, Hadat, Lebanon
6. Institut National de Santé Publique, Epidémiologie Clinique et Toxicologie (INSPECT-LB), Beirut, Lebanon
7. Department of Primary Care and Population Health, University of Nicosia Medical School, 2417, Nicosia, Cyprus
8. Neuroscience Research Center, Lebanese University, Faculty of Medical Sciences, Lebanon.

Abstract:

Background: Traffic and driving situations constitute a typical context that triggers anger among drivers which can lead to road traffic crashes (RTCs).

Objectives: This study aims to validate the Arabic version of the Driver Anger scale (DAS) and explore its multidimensional structure for assessing anger among Lebanese drivers. Besides, it targets to compare DAS to that of drivers from other countries and investigate the association between DAS and risk involvement (RTCs and traffic offenses).

Methods: Using a self-completion Arabic questionnaire, a cross-sectional study was conducted among Lebanese drivers recruited from all Lebanese governorates using a non-probability convenience sampling method. A face-to-face approach was used for data collection. The questionnaire includes demographic information and driving variables in addition to the Arabic version of DAS measured by 33 items. Data entry and statistical analysis were performed using SPSS version 24. Exploratory factor and confirmatory analysis were performed to investigate the factorial structure of the DAS. To test the correlation between continuous variables, the Pearson correlation test was used.  A comparison of DAS among Lebanese drivers with other countries was performed using independent-sample t-tests with summary data. Logistic regression analyses were performed to examine the association between DAS subscales and being involved in RTCs or being fined for traffic offenses.

### Results: A total of 1102 drivers participated in this study. The Exploratory Factor Analysis on DAS data differentiated six dimensions of driving anger with good reliabilities: hostile gestures (α=0.92), illegal driving (α=0.87), traffic obstruction (α=0.90), slow driving (α=0.92), discourtesy (α=0.81), and police presence (α=0.91). The results of the Confirmatory Factor Analysis demonstrated that the original six-factor model of the DAS fitted the Lebanese driving anger data. Gender, occupation, experience, and annual mileage differences were revealed in regards to anger subscales. Compared to other countries, Lebanese drivers displayed a high level of anger in the majority of anger aspects and the hostile gestures dimension was found the greatest contributing factor to driving anger. Drivers who experienced anger resulting from illegal driving, discourtesy, slow speeding, and police presence reported prior involvement in RTCs than their counterparts who didn’t experience such emotions.

### Conclusion: The Arabic version of the DAS is a valid and reliable tool for assessing traits driving anger among Lebanese drivers in different driving situations. The revealed role of driving anger in increasing the risk of RTCs in this study stressed the importance of adopting countermeasures to relieve driver anger.

Keywords: Driver anger scale (DAS), validation, adaptation, Road traffic crashes, traffic fines, Lebanese drivers

## Background:

Road traffic crashes (RTC), are one of the leading causes of unnatural death worldwide [1, 2]. Human factors related to risky behaviors adopted by road users, especially drivers [3-5] are among the main factors leading to RTCs. Given that driving style is a basic concept for road safety, researchers have expressed a particular interest [6] in exploring different driver behaviors and personality traits that might impact risky driving behavior and lead to RTCs [7-9]. Hence, they targeted many factors associated with dangerous driving behaviors including stress [10, 11], fatigue [12], personality traits [13-16], and driving anger as well, due to its high prevalence in the general population [17, 18].

Anger is a common feeling experienced in daily life due to multiple vexations and provocations conceived as perils to the individual's safety, or personal goals and values [19]. However, its presence is not essential or sufficient for the occurrence of human aggression and violence. It was defined as ‘an emotional state or condition characterized by feelings that range in intensity from the feeling of tension, mild irritation or annoyance to intense fury and rage [20] that may elucidate a variety of risk behaviors [7].

Driver anger was conceptualized as a personality trait related to an individual’s underlying predisposition for anger, but specific to road situations [21]. However, common driving situations, such as heavy traffic, impeding events, insolence, and diverted behavior of other road users could prompt intense levels of anger among anger-prone drivers with high traits of anger ( [22]. In the main, high trait of driving anger has been identified as a precursor of aggressive driving behaviors and high-level risk-taking in their driving style, which in turn increases the probability of being involved in RTCs [23-26]. Anger while driving can interfere with driving abilities such as attention, perception, information processing, and motor performance, which may cause an accident directly or indirectly.

To address the question of whether anger was more situational or contextual for some people, and as a result of extensive research, Deffenbacher et al. (1994) [21] developed a 33-item scale (long form), named the Driving Anger Scale (DAS), with a latent structure of six correlated dimensions (Deffenbacher et al., 1994): hostile gestures, illegal driving, police presence, slow driving, discourtesy, and traffic Obstructions [21]. These six subscales have been reported to show generally positive correlations with each other, which indicate a general trend for driving anger. Indeed, DAS is one of the most frequently used measurements for assessing traits driving anger and it has been tested among drivers from various countries such as the United Kingdom [27], New Zealand [28], Spain [29], France [30], Australia [31], Turkey [32], China [33], Japan [34], Poland [35], and Malaysia [36]. Despite that the original factorial structure of the DAS suggested by Deffenbacher et al. (1994)[21] has been confirmed in some studies with slight adjustments of the number of items [28, 32, 33, 36], this structure varies between different driving cultures and required specific adaption. For example, in the United Kingdom, a structure of three components (reckless driving, direct hostility, and impeded progress) was found [27], while the discourtesy component was removed from the structure in France [30]. Furthermore, validation studies have found different factor structures for the DAS suggesting that the dimensions of trait driving anger may also be context-dependent.

In all cases, it is not surprising that studies using this scale showed that individuals who had a high score in driving anger (when compared to those who scored low) are more likely and more intensively to be engaged in aggressive and risky behaviors [9, 37, 38] and were, therefore, more involved in road accidents.

Although anger is a universal emotion, anger-triggering situations heavily depend on the cultural context [32]. For a long time, Lebanon has been considered an unstable country. Heated displays of emotions are common in Lebanese daily life. Tempers of individuals often flare up easily over small things. Traffic and driving situations could be a typical context that triggers anger among road users, particularly among drivers. However, in a small country such as Lebanon, the current situation of the human epidemic of road traffic injuries (RTI) is alarming, the country witnessed a sustained increase in RTI due to traffic accidents. According to the Ministry of the Interior, 487 deaths and 6101 injuries resulted from RTCs, mainly due to human factors were recorded [39]. However, it remains unknown whether these driving behaviors are anger-related. To the best of our knowledge, no previous study in Lebanon has targeted the driving anger among Lebanese drivers For these reasons, it is of great interest to use DAS as a valuable tool in identifying drivers prone to anger, understanding what makes drivers angry, how serious their anger is in that situation and how this emotion condition subsequently contributes to accident involvement. Hence, there is a need to validate DAS in the Lebanese context and to understand how and to what DAS and its dimensions influence driver behavior and risk involvement (accident involvement and receiving traffic tickets).

The present study is, therefore, the first study in Lebanon that aimed to adapt and validate an Arabic version of the Driver Anger scale and to evaluate the propensity of Lebanese drivers to become angry. Besides it aims to examine, how socio-demographic characteristics affect driving anger and to investigate the association between DAS and the involvement in road accidents. Lastly, we want to contribute to a broader understanding of driving anger by comparing the Lebanese driver’s driving anger to that of drivers from other countries.

## Methodology:

The adaptation and validation of The Arabic version of DAS consisted of two stages. The first stage was the translation and cross-cultural adaptation of the long version of DAS(33 items) into Arabic and the second stage was the testing of the psychometric properties of the Arabic version of the DAS among Lebanese drivers.

The translation of the original 33-items version of DAS was meticulously performed following the forward-backward translation guidelines including translation synthesis [40]. The original 33-items version of DAS was translated from English to the Arabic language by two independent bilingual translators whose mother tongue is Arabic and who were also proficient in English. Inconsistencies found between the two translators were discussed and the two translated versions were synthesized into one. Then, the initial translated version was back-translated by two independent translators who are native speakers of the English language and who didn’t have previous knowledge about the original version of DAS. A committee of experts was composed to identify and verify linguistic, problematic items and discrepancies in terms of wording, and ambiguity of the DAS. Any suggested linguistic change to be made to the translated version was resolved by consensus. TA consensus was reached on keeping all the DAS items leading to a pre-final version of the translated DAS which is piloted on a small sample of 35 drivers.

Based on the feedback of respondents participating in the pilot testing, minor revisions including the change of ambiguous wording to the slang language were made to address potentially misleading items. Finally, the Arabic version of DAS was produced and ready for psychometric testing.

**Psychometric testing of DAS:**

### Study design and participants

This cross-sectional study is part of a large project exploring driver behavior in Lebanon. It was conducted from October to December 2019 among Lebanese drivers aged 18 years old and above recruited from all Lebanese provinces using a convenience sampling technique. A weighting procedure was adopted using predetermined target figures across gender and age for each Lebanese governorate (Bekaa, Baalbeck-Hermel, Mount-Lebanon, Beirut, North, Akkar, South, and Nabatyeh). to minimize sampling bias related to the overmentioned sampling technique. This can improve the representativeness of the sample in terms of age, gender, and location and align the sample distribution with the population for those variables.

All Lebanese active drivers having 18 years or over, having a driving license, driving regularly, and agreeing to participate were eligible to be part of this study. This study excluded drivers who are not practicing driving activities currently, or rarely drive (less than one time per month) after obtaining a driving license. Illiterate drivers who could not understand the questions and drivers who refused to participate in the study were also excluded. The research protocol was properly reviewed and approved by IPNET. All methods were performed following the relevant guidelines and regulations [41]. The study design assured adequate protection of study participants, and neither included clinical data about patients nor configured itself as a clinical trial.

### Minimal sample size calculation

The original version of the DAS scale consisted of 33 items therefore, 330 subjects were needed for performing the exploratory factor analysis based on Comrey and Lee's sample size guidance (5 to 10 subjects for each item) for establishing sufficient evidence of scale validity and reliability [42]. To increase the validity of the study, we used two different samples: one for the exploratory factor analysis (EFA) and one for the confirmatory factor analysis (CFA). In addition, we set to recruit more than 500 participants for each analysis (exploratory and confirmatory) to increase the study power and to manage losses related to withdrawal, or protocol violation.

### Reliability

The internal consistency reliability was estimated using Cronbach’s alpha where its value α ≥ 0.70 was considered satisfactory[43]. For the test‑retest reliability, 40 subjects were asked to fill out the questionnaire for the second time after almost three weeks, this duration between the first test and the retest aimed to avoid artificial reliability resulting from memory bias. Test-retest reliability was evaluated using Pearson correlation ((Pearson’s r) where its value ≥ 0.70 was considered satisfactory for ruling on the correlation between the retest and the initial study.

### Validity

The content validity of the DBQ was assessed using the viewpoints of the panel of eight experts including content experts as well as lay experts through a quantitative approach[44] . The experts rated each item of the DAS from 1 to 3 with a three-degree range of “not necessary, useful but not essential, essential”‏ respectively for assessing “driver anger”. This was quantified by the content validity‏ ratio (CVR) which was calculated using the following formula: $CVR=\frac{Ne-\frac{N}{2}}{\frac{N}{2}}$ .“Ne” referred to the number of experts who rated an item as “relevant.” And N referred to the total number of experts [45]. Assessment of the item aspects, in terms of the level of difficulty, ambiguity, necessary, and relevant items in the questionnaire are performed using the method proposed by the Lawshe method. (42 ) where a CVR is equal to or larger than 0.49 for each item indicating an acceptable level of significance, therefore‏ the item will be retained The Content Validity Index (CVI) is the mean score of those retained items having CVR≥0.49 [46].

As for construct validity, the two tests of Kaiser-Meyer-Olkin (KMO) and Bartlett were performed before factor analysis. The original sample was split into 2 samples containing approximately half of the participants. To determine whether the original DAS, including 33 items, was valid for the Lebanese population, and to identify drivers’ anger dimensions, the first sample (N=568), was subjected to Principal Component Analysis, and the items were exposed to factor analysis with Varimax rotation. Based on Eigenvalues >1 and scree plot, it was decided on the number of factors to be included in the model. Then, we performed a parallel analysis (PA) to determine the number of components or factors to retain from factor analysis. To evaluate the internal consistency of the DAS, Cronbach’s alpha reliability coefficients were calculated.

Confirmatory Factor Analyses (CFA) were performed using IBM AMOS 24.0. Fit indices and the respective cut-off for the goodness of fit were reported. The structural models were considered a good fit to the data when: the Chi-squared value (χ 2 )/degree of freedom (χ2/df < 5)[47], the Comparative Fit Index (CFI>0.9), the Tucker Lewis index (TLI) (a cut-off of 0.95 or greater stands for a good model fit), the Root Mean Square Error of Approximation (RMSEA<0.08), and the Standardized Root Mean Square Residual (SRMR<0.08) [46, 48]. In case of a poor fit, modification indices were examined to identify additional parameters that could be done to improve the goodness of fit of the models. Covariances were permitted to be freely estimated and items that loaded 0.40 on two or more factors (cross-loading items) were eliminated in the modified model (Byrne, 2006) [49].

**Data collection tool:**

An anonymous, Arabic, self-reported questionnaire was developed. It consisted of closed-ended questions and four sections. The first section included a brief introduction of the study objectives and instructions on how to fill out the questionnaire in addition to the informed consent, confirming the approval of the drivers to fill the questionnaire. The second section included questions assessing demographic and exposure measures. Participants were asked about their age, education level, marital status, working status, driving experience, driving frequency, and annual mileage. The third section covered the topic of risk involvement. Drivers were queried about the number of crashes (personal injury or property damage) and the number of fines they had within the past three years. The fourth section includes the overmentioned Arabic version of the DAS which consisted of 33 items. Participants were asked to rate how angry they would become if they came across each situation described by each item. Ratings were given based on a five-point Likert scale (1=not angry, 2= slightly angry, 3 = angry, 4=very angry 5=extremely angry). Higher scores reflected a high level of anger. None of the survey’s queries questioned for information that could harm the respondent in any way.

**Data collection procedure:**

After being screened for eligibility, participants were face to face approached by well-trained data collectors who were responsible to recruit participants from work, universities, parking stations, and public spaces in each Lebanese governorate. Before their enrollment in the study, potential subjects were informed about the study's purpose. After getting their acceptance to participate through written informed consent, data collectors delivered to them the instructions needed for filling out the questionnaire. No remuneration was given to the drivers for their contribution to the study which was entirely voluntary. Drivers were also free to withdraw from the study at any time. The completion of the questionnaire took around 10–15 minutes.

#### Statistical analysis

The collected data was entered and analyzed using the statistical software SPSS (Statistical Package for Social Sciences), version 24.0. Descriptive statistics were reported using frequency with percentages for categorical variables and mean and standard deviation for continuous variables. Since missing data constituted < 10% of the total database, then it was not substituted. Before running the analysis, the distribution of each DAS item was checked for normality. A bivariate analysis was conducted using the ANOVA test to compare the means of the DAS subscales and the categorical variables (socio-demographic variables). The effect size d is presented with 0.20 < d < 0.50 being a small effect, 0.50 < d < 0.80 being a medium effect, and 0.80 < d being a large effect [50]. Additionally, an international comparison of driving anger was performed using independent-sample t-tests with summary data. Pearson correlation was used for linear correlation between continuous variables. All variables that showed a *p*-value< 0.2 in the bivariate analysis were included in the model as independent variables. Multivariable logistic analyses were performed to examine the association between risk involvement (RTCs and fines) considered as dependent variables and DAS subscales as independent variables. The statistical significance level was set at a *p-value* < 0.05.

## Results

**Translation and content validity**

After evaluating the translation and back-translation of the Arabic version of DAS, as well as testing the pre-final version on 40 drivers, slight changes have been made, including the substitution of some confusing words, to produce the final version of DAS. Of note, the back-translated version matched closely the original one. Since all DAS items obtained a CVR of more than 0.75 based on the quantitative approach used by experts, the 33 items were retained. Moreover, the overall Content Validation Index (CVI) of DAS is 0.83. Therefore, the panel of experts considered the instrument appropriate to measure the driver’s anger and judged that the questionnaire had good content validity.

### Baseline characteristics of the participants

A total of 1102 drivers participated in this study. The majority of them were married (52.3%), aged less than 40 years old (66.4%), and holding a university degree or above (57.2%). The average number of years of driving experience was 13.46 (D=10.76). In terms of annual mileage, more than 50% of respondents drove more than 6000 kilometers annually. In terms of frequency of driving, the majority (62%) reported that they drive on daily basis and only 1.12% drove less than once monthly. Of note, 43.2% of surveyed drivers were involved in RTCs and 46.8% of them got penalized for traffic offenses in the previous 3 years.

### Driver Anger Scale-Arabic Version (DAS-A) items

Table 1 summarizes the mean values and standard deviations for each item of the DAS-33. All situations described in the scale provoked considerable amounts of anger among drivers. Of note, the highest level of anger reported was related to situations showing hostile gestures executed by other drivers such as obscene signs, honking, and yelling about their driving performance. On the contrary, items describing situations where the police are present (police monitoring, being pulled over, passing by a radar speed trap, or driving close to a police car) triggered the lowest level of anger. Both males and females reported anger while driving in these situations with slight fluctuations among gender.

**Table 1: Driver anger scale items mean and standard deviation**

|  |  | **Total** | | **Male** | | **Female** | |
| --- | --- | --- | --- | --- | --- | --- | --- |
|  |  | **Mean** | **S.D** | **Mean** | **S.D** | **Mean** | **S.D** |
| **DAS1** | Someone in front of you does not start up when the light turns green | 3.07 | 0.861 | 3.06 | 0.853 | 3.10 | 0.879 |
| **DAS2** | Someone is driving too fast for the road conditions | 3.64 | 0.557 | 3.65 | 0.542 | 3.63 | 0.592 |
| **DAS3** | A pedestrian walks slowly across the middle of the street, slowing you | 3.06 | 0.858 | 3.05 | 0.846 | 3.08 | 0.884 |
| **DAS4** | Someone is driving too slowly in the passing lane holding up traffic | 2.98 | 0.929 | 2.97 | 0.891 | 3.01 | 1.011 |
| **DAS5** | Someone is driving very close to your rear bumper | 3.36 | 0.699 | 3.33 | 0.684 | 3.42 | 0.729 |
| **DAS6** | Someone is weaving in and out of traffic | 3.69 | 0.705 | 3.67 | 0.709 | 3.73 | 0.695 |
| **DAS7** | Someone cuts in right in front of you on the motorway | 3.30 | 0.836 | 3.29 | 0.837 | 3.35 | 0.834 |
| **DAS8** | Someone cuts in and takes the parking spot you have been waiting for | 3.25 | 0.781 | 3.21 | 0.755 | 3.33 | 0.831 |
| **DAS9** | Someone is driving slower than reasonable for the traffic flow | 2.98 | 0.862 | 2.97 | 0.873 | 2.99 | 0.842 |
| **DAS10** | A slow vehicle on a mountain road will not pull over and let people by | 3.02 | 0.958 | 3.02 | 0.951 | 3.01 | 0.977 |
| **DAS11** | You see a police car watching traffic from a hidden position. | 2.30 | 1.071 | 2.34 | 1.111 | 2.19 | 0.970 |
| **DAS12** | Someone backs right out in front of you without looking | 3.27 | 0.778 | 3.25 | 0.775 | 3.34 | 0.783 |
| **DAS13** | Someone runs a red light or stop sign | 3.69 | 0.686 | 3.70 | 0.660 | 3.68 | 0.743 |
| **DAS14** | Someone coming toward you at night does not dim their headlights | 3.15 | 0.747 | 3.16 | 0.747 | 3.13 | 0.748 |
| **DAS15** | At night someone is driving right behind you with bright lights on | 3.34 | 0.913 | 3.31 | 0.900 | 3.39 | 0.944 |
| **DAS16** | You pass a radar speed trap | 2.39 | 1.031 | 2.45 | 1.069 | 2.26 | 0.930 |
| **DAS17** | Someone speeds up when you try to pass them | 3.37 | 0.842 | 3.34 | 0.831 | 3.44 | 0.866 |
| **DAS18** | Someone is slow in parking and holding up traffic | 3.02 | 0.963 | 3.02 | 0.954 | 3.01 | 0.986 |
| **DAS19** | You are stuck in a traffic jam | 3.34 | 0.871 | 3.36 | 0.876 | 3.31 | 0.861 |
| **DAS20** | Someone pulls right in front of you when there is no one behind you | 3.36 | 0.888 | 3.34 | 0.858 | 3.41 | 0.952 |
| **DAS21** | Someone makes an obscene gesture toward you about your driving | 3.81 | 0.679 | 3.79 | 0.661 | 3.85 | 0.718 |
| **DAS22** | You hit a deep pothole that was not marked | 3.38 | 0.797 | 3.38 | 0.800 | 3.39 | 0.792 |
| **DAS23** | A police car is driving in traffic close to you | 2.29 | 1.041 | 2.34 | 1.075 | 2.19 | 0.954 |
| **DAS24** | Someone honks at you about your driving | 3.79 | 0.665 | 3.78 | 0.654 | 3.82 | 0.691 |
| **DAS25** | Someone is driving well above the speed limit | 3.69 | 0.685 | 3.70 | 0.661 | 3.66 | 0.738 |
| **DAS26** | Driving behind a truck which is material flapping around in the back | 3.24 | 0.721 | 3.22 | 0.689 | 3.27 | 0.789 |
| **DAS27** | Someone yells at you about your driving | 3.76 | 0.635 | 3.74 | 0.613 | 3.81 | 0.680 |
| **DAS28** | A motorcyclist is riding in the middle of the lane and slowing traffic | 3.38 | 0.822 | 3.35 | 0.829 | 3.44 | 0.805 |
| **DAS29** | A police officer pulls you over | 2.35 | 1.062 | 2.41 | 1.106 | 2.23 | 0.949 |
| **DAS30** | You are behind a vehicle that is smoking badly (diesel fumes) | 3.33 | 0.910 | 3.34 | 0.907 | 3.30 | 0.917 |
| **DAS31** | A truck kicks up sand or gravel on the car you are driving | 3.46 | 0.953 | 3.48 | 0.962 | 3.39 | 0.932 |
| **DAS32** | You are driving behind a large truck and cannot see around it | 3.34 | 0.852 | 3.35 | 0.866 | 3.32 | 0.823 |
| **DAS33** | You encounter road construction and detours | 3.20 | 0.674 | 3.20 | 0.656 | 3.19 | 0.712 |

### Factor analysis

KMO test result (KMO = 0.891) was satisfactory indicating good sampling and Bartlett’s test was highly significant (p<0.001). As a result of factor analysis, using Varimax rotation, items converged over a solution of six factors that had eigenvalues over 1.0 and the examination of the scree plot suggested that the six-factor solution was the most interpretable one (Annex F1). Of note, the total variance explained was 88.6%, and Cronbach’s alpha = 0.93. Parallel analysis (PA) informed us also that four factors surpassed the PA criterion, which explained also 88% of the total variance. Both analyses yielded the same results in terms of the higher factor coefficient for each of the items selected. According to the Varimax rotated matrix, the loadings of the 33 items on each of these four factors are presented in Table 2. The items for each factor were similar to those in the original scale. As a result, the first factor with nine items was called ‘‘discourtesy”, accounting for the variance of 35%, and had an eigenvalue of 11.56 (α =0.81). The first factor explains 35% of the total variance. The second factor, called “traffic obstruction” included seven items which was responsible for 14.58% of the total variance (α=.90) and had an eigenvalue of 4.813. These items describe traffic barriers that impede drivers’ circulation. Factor 3, called “slow driving”, was comprised of six items. It was responsible for 13.7% of the total variance (α =0.92) and had an eigenvalue of 4.538. As for factor 4, it included four items and was labeled as “police presence”. It accounted for 10.6% (α =0.85) of the total variance and had an eigenvalue of 3.5. Factor 5, termed ‘‘Illegal driving”, included four items, accounted for 8.1% (α=0.87) of the total variance, and had an eigenvalue of 2.68. Factor 6, labeled “hostile gesture”, was composed of three items, was responsible for 6.48% (α=0.92) of the total variance and had an eigenvalue of 2.1.

**Table 2: Six-factor solution of DAS items, eigenvalues, Cronbach’s alpha coefficients, and variance explained by each DAS subscale among Lebanese drivers**

|  |  | **DAS factors** | | | | | |
| --- | --- | --- | --- | --- | --- | --- | --- |
|  |  | **Discourtesy** | **Traffic obstruction** | **Slow driving** | **Police presence** | **Illegal driving** | **Hostile gesture** |
| **DAS8** | Someone cuts in and takes the parking spot you have been waiting for | 0.835 |  |  |  |  |  |
| **DAS20** | Someone pulls right in front of you when there is no one behind you | 0.827 |  |  |  |  |  |
| **DAS14** | Someone coming toward you at night does not dim their headlights | 0.816 |  |  |  |  |  |
| **DAS15** | At night someone is driving right behind you with bright lights on | 0.808 |  |  |  |  |  |
| **DAS12** | Someone backs right out in front of you without looking | 0.807 |  |  |  |  |  |
| **DAS17** | Someone speeds up when you try to pass them | 0.776 |  |  |  |  |  |
| **DAS28** | A motorcyclist is riding in the middle of the lane and slowing traffic | 0.753 |  |  |  |  |  |
| **DAS7** | Someone cuts in right in front of you on the motorway | 0.709 |  |  |  |  |  |
| **DAS5** | Someone is driving very close to your rear bumper | 0.631 |  |  |  |  |  |
| **DAS33** | You encounter road construction and detours |  | 0.855 |  |  |  |  |
| **DAS26** | You are driving behind a truck that is material flapping around in the back |  | 0.852 |  |  |  |  |
| **DAS19** | You are stuck in a traffic jam |  | 0.833 |  |  |  |  |
| **DAS32** | You are driving behind a large truck and cannot see around it |  | 0.831 |  |  |  |  |
| **DAS22** | You hit a deep pothole that was not marked |  | 0.822 |  |  |  |  |
| **DAS31** | A truck kicks up sand or gravel on the car you are driving |  | 0.795 |  |  |  |  |
| **DAS30** | You are behind a vehicle that is smoking badly or giving off diesel fumes |  | 0.632 |  |  |  |  |
| **DAS18** | Someone is slow in parking and holding up traffic |  |  | 0.846 |  |  |  |
| **DAS10** | A slow vehicle on a mountain road will not pull over and let people by |  |  | 0.839 |  |  |  |
| **DAS4** | Someone is driving too slowly in the passing lane holding up traffic |  |  | 0.834 |  |  |  |
| **DAS9** | Someone is driving slower than reasonable for the traffic flow |  |  | 0.831 |  |  |  |
| **DAS2** | A pedestrian walks slowly across the middle of the street, slowing you |  |  | 0.817 |  |  |  |
| **DAS1** | Someone in front of you does not start up when the light turns green |  |  | 0.795 |  |  |  |
| **DAS23** | A police car is driving in traffic close to you |  |  |  | 0.850 |  |  |
| **DAS16** | You pass a radar speed trap |  |  |  | 0.845 |  |  |
| **DAS11** | You see a police car watching traffic from a hidden position. |  |  |  | 0.843 |  |  |
| **DAS29** | A police officer pulls you over |  |  |  | 0.938 |  |  |
| **DAS25** | Someone is driving well above the speed limit |  |  |  |  | 0.878 |  |
| **DAS6** | Someone is weaving in and out of traffic |  |  |  |  | 0.858 |  |
| **DAS2** | Someone is driving too fast for the road conditions |  |  |  |  | 0.857 |  |
| **DAS13** | Someone runs a red light or stop sign |  |  |  |  | 0.760 |  |
| **DAS21** | Someone makes an obscene gesture toward you about your driving |  |  |  |  |  | 0.829 |
| **DAS24** | Someone honks at you about your driving |  |  |  |  |  | 0.822 |
| **DAS27** | Someone yells at you about your driving |  |  |  |  |  | 0.808 |
|  | **Eigenvalue** | 11.560 | 4.813 | 4.538 | 3.501 | 2.689 | 2.141 |
|  | **Cronbach alpha** | 0.919 | 0.908 | 0.921 | 0.914 | 0.868 | 0.917 |
|  | **Variance** | 35.031 | 14.586 | 13.752 | 10.609 | 8.148 | 6.487 |
| ***Note:*** *88.6% of the variance was explained, Extraction Method: PCA, Rotation Method: Varimax with Kaiser Normalization.* | | | | | | | |

### Reliability analyses and inter-correlations between factors

Table 3 showed the reliability estimates. The α-values obtained of the subscales ranged between 0.87 and 0.93, indicating an excellent internal consistency. The overall reliability of the DAS-A scale was good (α=0.93). Similarly, all the subscales were reliable. Skewness [-0.21, 0.72] and kurtosis [-.1.08, 1.07] estimates for the four factors allowed the use of parametrical correlational analyses. Females ranked higher than males in the subscales of DAS related to discourtesy, illegal driving, and hostile gestures.

**Table 3: Summary statistics for the DAS subscales**

|  |  |  |  |  |  |  |  | **All drivers (n=1102)** | | | | | |
| --- | --- | --- | --- | --- | --- | --- | --- | --- | --- | --- | --- | --- | --- |
|  | # | **Number of items** | **Item mean** | **S.D** | **Min** | **Max** | **Scale means** | **S.D** | **Min** | **Max** | **α** | **Skewness** | **Kurtosis** |
|  | **DAS subscales** |  |  |  |  |  |  |  |  |  |  |  |  |
| D1 | Discourtesy | **9** | 3.34 | 0.82 | 1.4 | 4.89 | 30.09 | 7.51 | 13 | 44 | 0.81 | -0.21 | -0.94 |
| D2 | Traffic obstruction | **7** | 3.39 | 0.74 | 2.0 | 7.57 | 23.91 | 5.15 | 14 | 53 | 0.90 | 0.72 | 0.97 |
| D3 | Slow driving | **6** | 3.01 | 0.92 | 1.0 | 5.00 | 18.29 | 5.54 | 6 | 30 | 0.92 | 0.18 | -0.8 |
| D4 | Police presence | **4** | 2.33 | 1.04 | 1.0 | 4.50 | 9.37 | 4.16 | 4 | 18 | 0.85 | 0.24 | -0.98 |
| D5 | Illegal driving | 4 | 3.62 | 0.56 | 2.5 | 4.75 | 14.53 | 2.28 | 10 | 20 | 0.87 | 0.27 | -0.67 |
| D6 | Hostile gesture | **3** | 3.79 | 0.63 | 2.3 | 5.00 | 11.38 | 1.91 | 7 | 15 | 0.92 | 0.27 | -0.67 |
|  | **DAS scale** | **33** | 3.24 | 0.50 | 2.15 | 4.70 | 107.54 | 16.78 | 2.15 | 4.7 | 0.93 | 0.37 | -0.3 |
| **N.B:** S.D refers to standard deviation, alpha referred to Cronbach alpha, min for minimum, and max for maximum | | | | | | | | | | | | | |

### 2.3. Confirmatory factor analysis

The default model corresponding to the result of exploratory analysis doesn’t fit our data. Several model specifications were performed to achieve a better fit. Since DAS32 and DAS19 were highly correlated, DAS32: “You are behind a large truck and cannot see around it” was removed. We also covaried the items as follow: item 11 and 23, items 1 and 3, and items 20 and 17 (Annex F2).

DAS11: “You see a police car watching traffic from a hidden position” and DAS23:” A police car is driving in traffic close to you”. It is recommended that they can be merged into one item “Being watched by police”

DAS1: Someone in front of you does not start up when the light turns green and DAS3: A pedestrian walks slowly across the middle of the street, slowing you

DAS17: “Someone speeds up when you try to pass them” and DAS20 Someone pulls right in front of you when there is no one behind you. The model used in confirmatory factor analyses is presented schematically in figure 2. This resulted in a better fitting that the default model. The model fit measures of the data analysis were as follow χ2=2013.9, χ2/df =4.526 <5; NFI = 0.941>0.9, CFI = 0.953>0.9, TLI=0.948>0.9 GFI = 0.906>0.9, AGFI =0.902>0.9, RSMR=0.0469<0.08, RMSEA =0.071 <0.08 therefore suggesting a reasonable model fit (Annex T2).

### **International comparison of driving anger**

Table 4 summarized the comparison of the mean values for the DAS subscales between Lebanese drivers and drivers from other countries. In broad-spectrum, the mean values of the subscales obtained from Lebanese drivers were compatible with those obtained from American and Turkish drivers. The “police presence” subscale was the lowest anger trigger among Lebanese drivers compared to other subscales. Although the discourtesy subscale ranked among the top anger triggers in driving situations by drivers from different countries, this was the case for Lebanese drivers who get angry the most when exposed to hostile gestures. Of note, Lebanon ranked the top among listed countries in terms of anger related to hostile gestures (M=3.79), traffic obstruction (M=3.39), and illegal driving(M=3.62). Table 4 also indicates that the subscale with the highest mean score for the Lebanese sample was the hostile gestures subscale (M = 3.79), followed closely by the illegal driving subscale (M = 3.62). As in the New Zealander, Chinese and Spanish studies, anger provoked by police presence produced the lowest level of anger amongst the Lebanese drivers (M = 2.33).

After entering the means of the subscales of other countries into the SPSS syntax, t-tests were performed to compare Lebanese drivers with American, New Zealander, British, Spanish, Turkish, and Chinese drivers. The results indicate that Lebanese drivers reported significantly higher anger in terms of anger related to hostile gestures (3.79), traffic obstruction (3.39), and illegal driving(3.62) (p < .001) compared to other drivers from different countries. However, the discourtesy subscale was significantly lower among Lebanese drivers (p < .001) compared to American, New Zealander, German, American, and Turkish drivers but this level was similar to the one reported by Spanish drivers. Concerning the “police presence” subscale, Lebanese drivers have a similar level of anger to Chinese drivers. However, this level was significantly higher than the one (p < .001) reported by German, British, Spanish, and New-Zealanders drivers. Conversely, it is significantly lower than American drivers.

**Table 4: Mean scores of DAS factors from USA [21] (Deffenbacher et al. 1994), UK [27] (lajumen et al.1998), NZ [28](Sullman, 2006), Turkey [32](Yasak, 2008), Germany [51](Brandenburg 2021), China [33](Li 2014) versus Lebanon**

|  | **Subscale mean** | **Mean Difference** | | **T-test** | **P-value** | |
| --- | --- | --- | --- | --- | --- | --- |
| **Discourtesy** | | | | | | |
| Lebanon | **3.34** |  |  | | |  |
| Germany | 3.94 | -0.60 | -23.69 | | | <0.001 |
| United States of America | 3.90 | -0.56 | -22.10 | | | <0.001 |
| United Kingdom | 2.70 | 0.64 | 25.60 | | | <0.001 |
| New Zeeland | 3.50 | -0.16 | -6.20 | | | <0.001 |
| Turkey | 3.60 | -0.26 | -10.18 | | | <0.001 |
| Spain | 3.36 | -0.02 | -0.64 | | | **0.525** |
| China | 2.62 | 0.72 | 28.79 | | | <0.001 |
| **Traffic obstruction** | | | | | | |
| Lebanon | **3.39** |  |  | | |  |
| Germany | 3.28 | 0.11 | 6.17 | | | <0.001 |
| United States of America | 3.30 | 0.09 | 5.27 | | | <0.001 |
| United Kingdom | 2.00 | 1.39 | 63.87 | | | <0.001 |
| New Zeeland | 2.70 | 0.69 | 32.32 | | | <0.001 |
| Turkey | 2.77 | 0.62 | 33.22 | | | <0.001 |
| Spain | 3.10 | 0.29 | 0.76 | | | **0.446** |
| China | 2.70 | 0.69 | 33.22 | | | <0.001 |
| **Hostile gestures** | | | | | | |
| Lebanon | **3.79** |  |  | | |  |
| Germany | 3.34 | 0.45 | 23.55 | | | <0.001 |
| United States of America | 3.20 | 0.59 | 30.83 | | | <0.001 |
| United Kingdom | 2.30 | 1.49 | 77.64 | | | <0.001 |
| New Zeeland | 2.70 | 1.09 | 56.83 | | | <0.001 |
| Turkey | 2.68 | 1.11 | 57.87 | | | <0.001 |
| Spain | 3.40 | 0.39 | 20.43 | | | <0.001 |
| China | 2.68 | 1.11 | 57.87 | | | <0.001 |
| **Slow driving** |  |  |  | | |  |
| Lebanon | **3.01** |  |  | | |  |
| Germany | 2.77 | 0.24 | 10.04 | | | <0.001 |
| United States of America | 3.20 | -0.19 | -5.42 | | | <0.001 |
| United Kingdom | 2.00 | 1.01 | 37.73 | | | <0.001 |
| New Zeeland | 2.80 | 0.21 | 8.96 | | | <0.001 |
| Turkey | 2.32 | 0.69 | 26.22 | | | <0.001 |
| Spain | 2.90 | 0.11 | 5.37 | | | <0.001 |
| China | 2.39 | 0.62 | 23.70 | | | <0.001 |
| **Police Presence** |  |  |  | | |  |
| Lebanon | **2.33** |  |  | | |  |
| Germany | 2.13 | 0.20 | 6.84 | | | <0.001 |
| United States of America | 3.00 | -0.67 | -21.23 | | | <0.001 |
| United Kingdom | 1.40 | 0.93 | 30.39 | | | <0.001 |
| New Zeeland | 1.90 | 0.43 | 14.26 | | | <0.001 |
| Turkey | 2.00 | 0.33 | 11.04 | | | <0.001 |
| Spain | 2.20 | 0.13 | 4.58 | | | <0.001 |
| China | 2.32 | 0.01 | 0.71 | | | **0.476** |
| **Illegal driving** | | | | | | |
| Lebanon | **3.62** |  |  | | |  |
| Germany | 2.98 | 0.64 | 37.86 | | | <0.001 |
| United States of America | 2.70 | 0.92 | 54.14 | | | <0.001 |
| United Kingdom | 2.30 | 1.32 | 77.39 | | | <0.001 |
| New Zeeland | 3.30 | 0.32 | 19.26 | | | <0.001 |
| Turkey | 3.46 | 0.16 | 9.96 | | | <0.001 |
| Spain | 3.50 | 0.12 | 7.64 | | | <0.001 |
| China | 2.54 | 1.08 | 63.44 | | | <0.001 |

### The relationship between driving anger and its subscales

There were significant correlations [ranging from r =0.178 (p < 0.01) to r =0.510 (p < 0.01) between the subscales of DAS (Annex T1). As expected, there were moderate to highly positive correlations between the DAS scale and its 6 dimensions. The highest correlation was between the DAS scale and discourtesy (r=0.775, CI(0.591-0.812), p<0.01) showing a positive linear relationship followed by DAS and the slow driving component (r=0.702, CI(0.602-0.854), p<0.01).

### DAS-A and socio-demographic variables

Table 6 shows the mean anger reported for each of the six factors, as well as overall driving anger for the sociodemographic variable. The overall DAS varied between males and females. Hostile gestures and discourtesy were the behaviors that instigated the most anger in both males and females, and police presence resulted in the least. Males and females differed in driving anger expressed toward hostile gestures (p=0.047), discourtesy (p=0.025 < .001) and illegal driving (p =0.002), police presence (p=0.034), and traffic obstruction (p=0.048) with female drivers rating discourtesy, hostile gesture, illegal driving and traffic obstruction as significantly more anger-provoking than male drivers did. However, police presence instigated more anger among males than females. Since Cohen’s d is small indicating a limited effect size, they may have occurred because of the large sample size. In terms of educational level, no differences regarding anger provoked by driving situations were revealed between drivers with higher educational levels and those with a lower ones.

On the other hand, the occupational difference was revealed between professional drivers and non-professional drivers in terms of anger prompted by police presence (p<0.001), traffic obstruction (p=0.034), and slow speeding (p<0.001) with professional drivers rating slow driving, police presence and traffic obstruction as significantly more anger-provoking than nonprofessional drivers did. In terms of annual mileage, drivers traveling larger distances (>6000 Km) prompted more anger related to illegal driving, discourtesy, traffic obstruction, and slow speeding. Of note, the DAS overall score revealed significant differences by gender, occupation, and annual mileage.

Pearson’s correlation coefficients were calculated to examine relationships between driving anger subscales as well as total overall anger with a driver’s age, and years of driving experience. As they get older, drivers report experiencing less driving anger as a result of discourtesy, and police presence. In contrast, driving anger flared in response to illegal driving (r=0.224), slow speeding(r=0.096) and traffic obstruction(r=0.06) increases with increasing age. Along with increased driving experience, driving anger experienced in reaction to discourtesy, slow driving, and illegal driving increased. However, anger instigated by police presence decreased with large experience (Table 5).

**Table 5: Driver anger scale (DAS-A) and socio-demographics characteristics**

|  | **Gender** | | | **F-value** | | |  | **P-value** | | | | | **Cohen's d** | | | |  |
| --- | --- | --- | --- | --- | --- | --- | --- | --- | --- | --- | --- | --- | --- | --- | --- | --- | --- |
|  | **Male (N=754)** | **Female (N=348)** | |  | | |  |  | | | | |  |  |  |  |  |
|  | **N(SD)** | **N(SD)** | |  | | |  |  | | | | |  | | | |  |
| **Hostile gesture** | 3.783(0.628) | 3.913(0.659) | | 3.520 | | |  | 0.047 | | | | | 0.22 | | | |  |
| **Illegal driving** | 3.306(0.832) | 3.527(0.836) | | 4.988 | | |  | 0.026 | | | | | 0.265 | | | |  |
| **Discourtesy** | 3.622(0.564) | 3.752(0.586) | | 3.638 | | |  | 0.025 | | | | | 0.226 | | | |  |
| **Police presence** | 2.386(1.051) | 2.246(0.975) | | 4.481 | | |  | 0.034 | | | | | 0.138 | | | |  |
| **Traffic obstruction** | 3.406(0.745) | 3.539(0.718) | | 2.468 | | |  | 0.048 | | | | | 0.182 | | | |  |
| **Slow speeding** | 3.027(0.923) | 3.085(0.923) | | 0.770 | | |  | 0.380 | | | | | 0.063 | | | |  |
| **DAS total** | 3.248(0.511) | 3.510(0.504) | | 11.153 | | |  | 0.028 | | | | | 0.51 | | | |  |
|  |  | | **Educational level** | | | | | | | | | | | | | |  |
|  | **Secondary or less (N=468)** | **More than secondary (N=634)** | |  | | |  | | | | | | |  |  | |  |
| **Hostile gesture** | 3.787(0.657) | 3.796(0.624) | | 0.051 | | |  | | 0.822 | | | | 0.02 | | | |  |
| **Illegal driving** | 3.651(0.583) | 3.617(0.563) | | 0.928 | | |  | | 0.336 | | | | 0.06 | | | |  |
| **Discourtesy** | 3.367(0.841) | 3.327(0.831) | | 0.606 | | |  | | 0.437 | | | | 0.05 | | | |  |
| **Police presence** | 2.403(1.063) | 2.296(1.001) | | 2.884 | | |  | | 0.090 | | | | 0.103 | | | |  |
| **Traffic obstruction** | 3.414(0.747) | 3.419(0.728) | | 0.008 | | |  | | 0.927 | | | | 0.01 | | | |  |
| **Slow speeding** | 3.089(0.950) | 3.020(0.903) | | 1.507 | | |  | | 0.220 | | | | 0.08 | | | |  |
| **DAS total** | 3.280(0.534) | 3.244(0.489) | | 1.353 | | |  | | 0.245 | | | | 0.07 | | | |  |
|  |  | | **Occupation** | | | | | | | | | | | | | |  |
|  | **Non-professional Driver (N=986)** | **Professional driver (N=116)** | |  | |  | | | | | | | |  |  | |  |
| **Hostile gesture** | 3.796(0.634) | 3.767(0.681) | | 0.208 | |  | | | | 0.649 | | | 0.04 | | | |  |
| **Illegal driving** | 3.634(0.562) | 3.614(0.643) | | 0.117 | |  | | | | 0.733 | | | 0.03 | | | |  |
| **Discourtesy** | 3.334(0.817) | 3.427(0.975) | | 1.287 | |  | | | | 0.257 | | | 0.104 | | | |  |
| **Police presence** | 2.299(0.817) | 2.700(1.001) | | 15.940 | |  | | | | <0.001 | | | 0.439 | | | |  |
| **Traffic obstruction** | 3.426(0.736) | 3.340(0.737) | | 1.418 | |  | | | | 0.034 | | | 0.12 | | | |  |
| **Slow speeding** | 3.016(0.919) | 3.337(0.911) | | 12.651 | |  | | | | <0.001 | | | 0.351 | | | |  |
| **DAS total** | 3.248(0.497) | 3.349(0.591) | | 4.139 | |  | | | | 0.042 | | | 0.19 | | | |  |
|  |  | | **Annual mileage** | | | | | | | | | | | | | |  |
|  | **≤6000 km (N=524)** | **>6000 Km (N=578)** | |  | |  | | | | |  | | |  | | |  |
| **Hostile gesture** | 3.782(0.607) | 3.803(0.666) | | 0.296 | |  | | | | | 0.586 | | | 0.02 | | |  |
| **Illegal driving** | 3.576(0.693) | 3.682(0.568) | | 9.507 | |  | | | | | 0.002 | | | 0.167 | | |  |
| **Discourtesy** | 3.185(0.817) | 3.488(0.825) | | 37.276 | |  | | | | | <0.001 | | | 0.369 | | |  |
| **Police presence** | 2.332(1.017) | 2.352(1.040) | | 0.104 | |  | | | | | 0.747 | | | 0.019 | | |  |
| **Traffic obstruction** | 3.363(0.715) | 3.465(0.752) | | 5.239 | |  | | | | | 0.022 | | | 0.14 | | |  |
| **Slow speeding** | 2.945(0.867) | 3.144(0.963) | | 12.814 | |  | | | | | <0.001 | | | 0.21 | | |  |
| **DAS total** | 3.177(0.477) | 3.333(0.525) | | 25.849 | |  | | | | | <0.001 | | | 0.311 | | |  |
|  | **Age** | **Pearson r** | | | **C.I.** | | | | | | | **P-value** | | | | | |
| **Hostile gesture** |  | 0.051 | | | | (-0.131;0.098) | | | | | | | | p>0.05 | |  |  |
| **Illegal driving** |  | 0.224^**^ | | | | (0.132;0.376) | | | | | | | | p<0.01 | |  |  |
| **Discourtesy** |  | -0.203^**^ | | | | (-0.344;-0.122) | | | | | | | | p<0.01 | |  |  |
| **Police presence** |  | -0.100^**^ | | | | (-0.298;-0.076) | | | | | | | | p<0.01 | |  |  |
| **Traffic obstruction** |  | 0.060^*^ | | | | (0.015;0.121) | | | | | | | | p<0.05 | |  |  |
| **Slow speeding** |  | 0.096^**^ | | | | (0.019-0.132) | | | | | | | | p<0.01 | |  |  |
| **DAS total** |  | 0.151 | | | | (-0.022;0.253) | | | | | | | | p<0.01 | |  |  |
|  | **Year of experience** | |  | | | | | | | | | | | | |  |  |
| **Hostile gesture** |  | 0.028 | | | | (-0.211;0.156) | | | | | | | | p>0.05 | |  |  |
| **Illegal driving** |  | 0.179^**^ | | | | (0.078;0.283) | | | | | | | | p<0.01 | |  |  |
| **Discourtesy** |  | 0.151^**^ | | | | (-0.344;-0.122) | | | | | | | | p<0.01 | |  |  |
| **Police presence** |  | -0.125^**^ | | | | (-0.202;-0.002) | | | | | | | | p<0.01 | |  |  |
| **Traffic obstruction** |  | 0.021 | | | | (-0.118;0.093) | | | | | | | | p>0.05 | |  |  |
| **Slow speeding** |  | 0.097^**^ | | | | (0.031-0.133) | | | | | | | | p<0.01 | |  |  |
| **DAS total** |  | 0.103^**^ | | | | (0.076;0.196) | | | | | | | | p<0.01 | |  |  |
| *N.B: ^**^ : p<0.01, ^*^ : p<0.05, C.I: Confidence interval* | | | | | | | | | | | | | | | |  |  |

### Association between Driver anger and road traffic crashes and fines

Table 6 displayed the multivariable logistic regression of the DAS subscales with the drivers' involvement in RTCs and being penalized for traffic offenses in the past 3 years. After controlling for potential confounders,  drivers who are more prone to experience anger resulting from illegal driving (aOR=1.771, 95% C.I (1.332-2.354)), discourtesy (aOR=1.806, 95% C.I (1.657-1.988), slow speeding (aOR=1.1663, 95%C.I (1.017-1.358) and police presence(aOR=1.341, 95% C.I (1.182-1.520) were more likely to have been involved in RTCs than their counterparts who didn’t experience such emotions. In terms of traffic fines, only drivers who were provoked by traffic obstruction (aOR=1.330, 95%C.I (1.108-1.5955)) were more likely to be penalized for traffic offenses.

### Table 6: Multivariable logistic regression of the DAS subscales with the drivers' involvement in RTCs and being penalized for traffic offenses in the past 3 years

|  | **Involvement in RTC** | | | | **Getting traffic offenses** | | | |
| --- | --- | --- | --- | --- | --- | --- | --- | --- |
|  | **P-value** | **aOR** | **95% C.I. for aOR** | | **P-value** | **aOR** | **95% C.I. for aOR** | |
|  |  |  | **Lower** | **Upper** |  |  | **Lower** | **Upper** |
| **Gender** | 0.056 |  |  |  | 0.984 |  |  |  |
| **Occupation** | <0.001 |  |  |  | <0.001 |  |  |  |
| Non-professional driver | Ref |  |  |  | Ref |  |  |  |
| Professional driver | 0.003 | 1.938 | 1.254 | 2.996 | 0.001 | 2.138 | 1.358 | 3.367 |
| **Annual mileage (km)** | <0.001 |  |  |  | <0.001 |  |  |  |
| ≤6000km | Ref |  |  |  | Ref |  |  |  |
| >6000km | <0.001 | 2.433 | 1.867 | 3.171 | <0.001 | 2.063 | 1.580 | 2.694 |
| **Age** | 0.035 | 0.786 | 0.589 | 0.802 | 0.540 |  |  |  |
| **Years of experience** | 0.016 | 0.812 | 0.732 | 0.934 | 0.530 |  |  |  |
| **Education level** (more than secondary vs less than secondary) | 0.145 |  |  |  | 0.055 |  |  |  |
| **Hostile gesture** | 0.123 |  |  |  | 0.655 |  |  |  |
| **Illegal driving** | <0.001 | 1.771 | 1.332 | 2.354 | 0.261 |  |  |  |
| **Discourtesy** | 0.038 | 1.806 | 1.657 | 1.988 | 0.737 |  |  |  |
| **Police presence** | <0.001 | 1.341 | 1.182 | 1.520 | 0.102 |  |  |  |
| **Traffic obstruction** | 0.693 | 0.964 | 0.801 | 1.159 | 0.002 | 1.330 | 1.108 | 1.595 |
| **Slow speeding** | 0.045 | 1.163 | 1.017 | 1.358 | 0.936 |  |  |  |
| **aOR: adjusted odds ratio, C. I confidence interval** | | | | | | | | |
|  | | | | | | | | |

### Discussion

The propensity to become angry behind the wheel (trait-driving anger) has been the subject of many studies focusing on how emotional and personality factors influence traffic behavior and accident involvement. The objective of the current research was to examine the psychometric properties of the Arabic version of DAS in terms of internal consistency reliability, and construct validity among Lebanese driver. To the best of our knowledge, this is the first national study in Lebanon that targets driving anger and adapting DAS. Hence, the Arabic version of the DAS may significantly contribute to this field of road user behavior in Lebanon and in other Arabic speaking countries. The further examination of the psychometric performance of the DAS in Lebanese drivers will enhance its validity.

The present study showed the DAS-A with its 33 items has satisfactory psychometric properties. Each factor loading was significant, with values greater than .60; and all six subscales had good to excellent reliability, with alpha coefficients greater than .80. The confirmatory factor analysis confirmed the original six-factor solution found by Deffenbacher et al., 1994 which provided a good fit to our data namely hostile gestures, illegal driving, discourtesy, traffic obstruction, police presence, and slow driving. The high correlations between the adapted Arabic version of DAS and its subscales for Lebanese drivers, the high alpha reliability, and the fit of the data to the factorial structure indicated that the Arabic version of DAS could be used to measure driving anger traits among Lebanese drivers and in further studies as well. The overall DAS score among Lebanese drivers was 107.5 which is considered high compared to other countrie and Lebanese drivers exhibited significantly higher anger towards situations including hostile gestures, traffic obstruction, and illegal driving compared to other drivers from different countries. Lastly, drivers who exhibited anger toward illegal driving, slow speeding and police presence were more likely to report prior involvement in RTCs than their counterparts who didn’t experience such emotions. In terms of traffic fines, only drivers who were provoked by traffic obstruction were more likely to be penalized for traffic offenses.

Our findings regarding the multidimensional structure of DAS were similar to the findings of Sullman et al. (2007) [28] and to those found in a study conducted among Chinese drivers [33]. Of note, some studies where some minor changes were made concerning the number of items have also confirmed the original factor structure of the DAS [28, 32, 33, 36]. However, this factorial structure was different from the findings of other studies such as the study conducted by Lajunen et al., 1998[52] that found a multifactorial structure consisting of only three factors rather than the six subscales . In New Zealand, a four-factor solution of DAS was found [28](and the discourtesy factor was deleted in France [30]. These differences among these factor structures could be related to methodological dissimilarities. Of note, the inter-correlations found in the present study among the six factors were at median level with a range from 0.178 to .610, suggesting these six subscales might be loaded on multiple construct.

In comparison with driving anger across samples of drivers from various countries, the overall DAS score among Lebanese drivers was 107.5 which is considered high. Our findings are comparable to the United States where the average score of DAS was 109.0 [53]. However, an average score for the complete DAS was only 69.2 among Japanese drivers [34]. The level of anger that drivers reported on the six subscales of the DAS differs from country to country.

The international comparisons of driving anger Based on the original factorial structure of DAS that the “police presence” subscale was the lowest anger trigger among Lebanese drivers. These results were compatible with those obtained from American [21], French [30], Turkish drivers [32], and Chinese drivers [33]. Concerning the hostile gestures subscale, it ranked as the top driving stimuli that provoked anger among Lebanese drivers. In opposition to other studies [21, 32, 33], discourtesy was not the major trigger of anger in Lebanese drivers. Our findings showed that Lebanese drivers get angry the most when exposed to hostile gestures. Remarkably, Lebanon ranked the top among Americans, and Turkish, in terms of anger related to hostile gestures (3.79), traffic obstruction (3.39), and illegal driving(3.62) compared to other countries [21, 32, 33, 53], . However, the discourtesy subscale was significantly lower among Lebanese drivers (p < .001) compared to American [21], New Zealanders [28], German [51], Chinese [33], and Turkish [32] drivers but this level was similar to the one reported by Spanish drivers [54]. As for the police presence subscale, Lebanese drivers have a similar level of anger to Chinese drivers. However, this level was significantly higher than the one (p <0.001) reported by German, British, Spanish, and New-Zealanders drivers [28, 29, 51, 54]. Conversely, it is significantly lower than American drivers. Lastly, levels of anger prompted by traffic obstruction among Lebanese and Spanish [54] drivers were similar. This could be explained by a cultural difference since the display of heated emotions are common in Lebanese daily life. It is well known that the tempers of the Lebanese population often flare up easily over small things such as traffic, yet such anger dissipates fairly quickly. This can explain the high level of anger among Lebanese drivers. Forthcoming studies that focus on the possible effects of culture on driving anger were recommended.

Since researchers have found that demographic variables affect a driver’s propensity to experience driving anger, we also focused in the present study on driving anger and its relationship to demographic variables such as age and gender. Our findings showed that the overall DAS varied between males and females. Gender differences were revealed in driving anger related to all DAS subscales except slow driving. Female drivers reported discourtesy, hostile gestures, illegal driving, and traffic obstruction as significantly more anger-provoking than male drivers did. However, police presence instigated more anger among males than females. Consistently to our results, Sullman et al., 2014 [36] found a significantly higher tendency to experience anger for females than for males in terms of traffic obstructions, and hostile gestures. Besides, Sullman et al., 2007 also found that females were more prone to become angry facing discourtesy, traffic obstructions, and illegal driving [54].

In terms of age, as they get older, drivers reported that they experienced less driving anger as a result of discourtesy and police presence. Our results were comparable to a Chinese study conducted by Li et al. (34) that found that younger drivers reported significantly higher levels of driving anger than drivers from older age groups did. Besides, a study conducted among Malaysian drivers conducted by Sullman et al. (2014) revealed a negative correlation between driving anger and age [36]. In another study, Sullman et al. (2007) [54] stated that older age was related to a lower tendency toward driving anger, concerning discourtesy, police presence, and overall driving anger. Of note, such a relationship between gender and age and a driver’s tendency toward driving anger was not found in other studies like in Japan and Germany [34, 51]. Hence, the extent of the relationship between driving anger and demographic variables like age and gender is still not completely distinct [22]. It is noteworthy that no differences regarding anger provoked by driving situations were revealed between drivers with higher educational levels and those with lower ones. On the other hand, the occupational difference was revealed between professional drivers and non-professional drivers in terms of anger prompted by the police presence, traffic obstruction, and slow speeding with professional drivers rating slow driving, police presence, and traffic obstruction as significantly more anger-prompting than nonprofessional drivers did. This disparity was also highlighted among professional drivers (taxi, bus, truck)[55].

In terms of annual mileage, drivers who traveled long distances expressed more anger related to illegal driving, discourtesy, traffic obstruction, and slow speeding. This could be explained that an extensive exposure to road situations could instigate higher level of anger especially the factors affecting the fluidity of the road (obstruction and speeding). However, increasing age was positively correlated to anger prompted by illegal driving, slow speeding, and traffic obstruction. However, In a study conducted among Malaysian drivers, age was found negatively related to five (discourtesy, traffic obstructions, hostile gestures, slow driving and police presence)[36]. Furthermore, several studies showed that age, anger and aggressive driving behavior were closely associated. Younger aged drivers correlated with more driving anger, risky, reckless and aggressive driving behaviors, which may be associated with an increased risk of traffic crash involvement[56-58].

Correspondingly to the driving experience increase, driving anger experienced in reaction to discourtesy, slow driving, and illegal driving increased. Our results were not consistent with the findings of a study that obtained a negative correlation between anger and years of a driver’s license possession and more experienced drivers showed less driving anger in response to events related to discourtesy. However, anger instigated by police presence decreased with large experience.

Our study also focused on DAS general and specific DAS subscale scores in predicting an external criterion (e.g. traffic tickets, traffic accidents), using the six factorial solutions. Drivers who are more prone to experience anger resulting from illegal driving, discourtesy, slow speeding, and police presence were more likely to be involved in RTCs than their counterparts who didn’t experience such emotions. Apart from that, this study also found that young drivers, less experienced drivers, long-distance driving, and drivers who were more likely to feel angry while driving reported prior involvement in road accidents. In terms of traffic fines, only drivers who were provoked by traffic obstruction were more likely to be penalized for traffic offenses. Our results were partially consistent with the findings of Sullman et al., where DAS dimensionsal were not directly associated to road crash involvement. However, several of the six forms of DAS were significantly associated to the crash-related conditions such as being ticketed [36].

### Limitations

Several limitations should be mentioned in our study, methodological limitations in particular. First, no causal inferences can be made from the results of this study between driving anger and different driving outcomes given the cross-sectional design of the study. A selection bias is possible due to the snowball technique used to recruit the sample which can sometimes provide quite homogenous sets of data. Second, the data collection was based solely on drivers’ self-report anger and no observations were made. This method is known to have two main limitations: social desirability and cognitive distortions. Participants could provide socially desirable responses. They were informed about the topic of the study and maybe they could have provided a “perfect driver profile” to meet the social norms. It should be noted, however, that the participants were volunteers and they were assured anonymity and confidentiality and would not benefit from embellishing their responses. Thus, taking into account the somewhat internalizing nature of driving anger and the fact that it could be difficult to measure it via observational methods, self-reports may represent a quite feasible approach. Third, the measurement of accident involvement and traffic fines was based on a self-report of past accidents and traffic offenses. Simply because of forgetting to report some accidents (Maycock et al., 1991), some respondents may have underestimated the number of RTCs and fines in which they had been involved. The target group of the present study was Lebanese drivers. Although refugees might represent a large proportion of the driver population in Lebanon, the present study was restricted to Lebanese drivers because of difficulties in data collection (e.g., difficulties to obtain representative samples). Lastly, despite the rich demographic measures, we could have missed some subgroups of the population and some factors associated with the driving anger propensity that could have changed our results, predisposing us to a residual confounding bias.

### Future research

Although the increasing research attention accounted for driving anger, additional research is required in this area to provide a more comprehensive and deeper understanding of driving anger and to support the improvement of intervention plans to eliminate the adverse effects of driving anger. Our study’s findings and its limitations show the importance of investigating the driver anger phenomenon with a mixed methodology combining qualitative and quantitative methods. First, studies using more representative samples of the general driver populations as well as more specific driver groups such as the elderly or professional drivers are required. Although the severity of road rage is increasing in eastern countries, the problem has not yet been given much attention. To appraise the effects of driving anger, future studies should consider using more objective measures (e.g. police reports or insurance records) of traffic accidents and fines. Finally, future studies focusing on state driving anger are needed for a deeper understanding of the mechanism of anger.

## Conclusion

In conclusion, this study has found that the Arabic version of the DAS is a valid and reliable tool for assessing trait driving anger in different driving situations and confirmed the original six-factor structure of DAS. In addition, hostile gestures were found to be the greatest contributing factor in causing Lebanese drivers to become angry while driving, followed closely by illegal driving. Compared to other countries, Lebanese drivers displayed a high level of anger in the majority of aspects. This study as well revealed the role of driving anger in increasing the risk of RTCs. To overcome this issue, the authorities and enforcement teams should be more focused on adopting countermeasures to relieve driver anger and decrease its negative impact. Advocating tolerance in driving style is also recommended.

**List of Abbreviations:**

RTC: Road traffic crashes

DAS: Driver Anger scale

DAS-A: Arabic version of the Driver Anger Scale

C.I: Confidence interval

aOR: Adjusted Odds Ratio

RTCs: Road traffic crashes

P: P-value

M: Mean

SD: Standard deviation

SPSS: Statistical Package for Social Sciences

CFA: Confirmatory factor analysis

EFA: Exploratory factor analysis

KMO: Kaiser–Meyer–Olkin

CVI: Content Validity Index

CVR: Content validity ratio

RMSEA: Root mean square error of approximation

SRMR: Standardized root mean square residual

GFI: Goodness-of-Fit Index

CFI: Comparative Fit Index

NFI: Normed Fit Index

DBQ: Driver behavior questionnaire

α: Cronbach’s alpha

r: coefficient de correlation r

d: Cohen’d

IPNET: Lebanese Higher Institute of Technical & Professional

### Declarations:

#### Ethical considerations

Written informed consent was obtained for each participant. They were reassured that their participation is voluntary and that they were free to withdraw at any time. In addition, all information was gathered anonymously and handled confidentially. The study design assured adequate protection of study participants, and neither included clinical data about patients nor configured itself as a clinical trial.

#### Funding

No funding was received.

#### Consent for publication

Not applicable.

#### Competing interests

The authors declare that they have no competing interests.

#### Acknowledgments

The authors acknowledge all drivers who participated in this study as well as the student of traffic major in IPNET Lebanese Higher Institute of Technical & Professional, Bir Hassan who helped us in the data collection.

#### Availability of data and materials:

The datasets used and analyzed during the current study are available from the corresponding author on reasonable request.

#### Authors’ contributions

Conception and design: D.Y

Analysis and interpretation of the data: D.Y and L.A.A

Drafting of the article: D.Y,

Critical revision of the article for important intellectual content: D.Y, P.S and LR.S

Final approval of the article: D.Y, P.S, L.A.A and L.R.S

#### Authors information

Dalal Youssef, MSc, MPH, RSM, Ph.D., Bordeaux Research Center for Population Health, Institut de santé publique, d'épidémiologie et de développement (ISPED), Bordeaux University, France & Clinical trial Program, Ministry of Public Health, Beirut, Lebanon & Lebanese Higher Institute of Technical & Professional, Bir Hassan, Ministry of Education, Beirut, Lebanon ORCID IDs: [https://orcid.org/0000-0003-3085-6849](https://orcid.org/0000-0003-3085-6849?lang=en) Email: [dyoussef@moph.gov.lb](mailto:dyoussef@moph.gov.lb); [dalalyoussef.esu@gmail.com](mailto:dalalyoussef.esu@gmail.com); Phone number: +96171239633

Pascale Salameh, PharmD, Ph.D., Lebanese university

Linda Abou Abbas, MPH, Ph.D., Ministry of Public Health, Epidemiological surveillance unit

Louis-Rashid Salmi, MD, Ph.D., ISPED Bordeaux

Annexes:

Annex F1


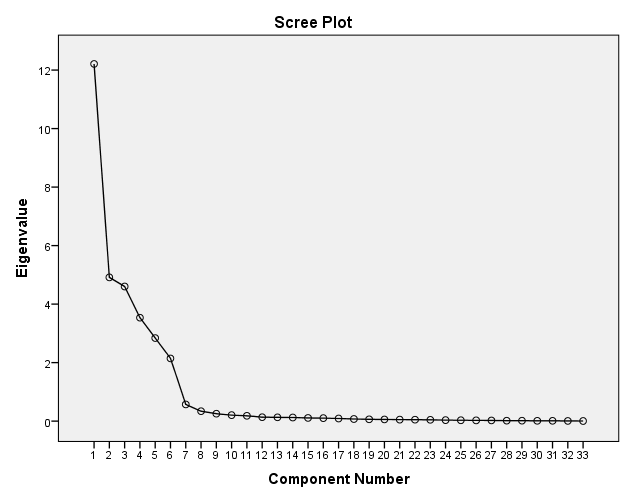


F1: Scree plot showing the number of factors in the Arabic version of DAS.

Annex F2: Factorial structure of Driver Anger Scale (DAS-A)

**Factorial structure of Driver Anger Scale (DAS)**


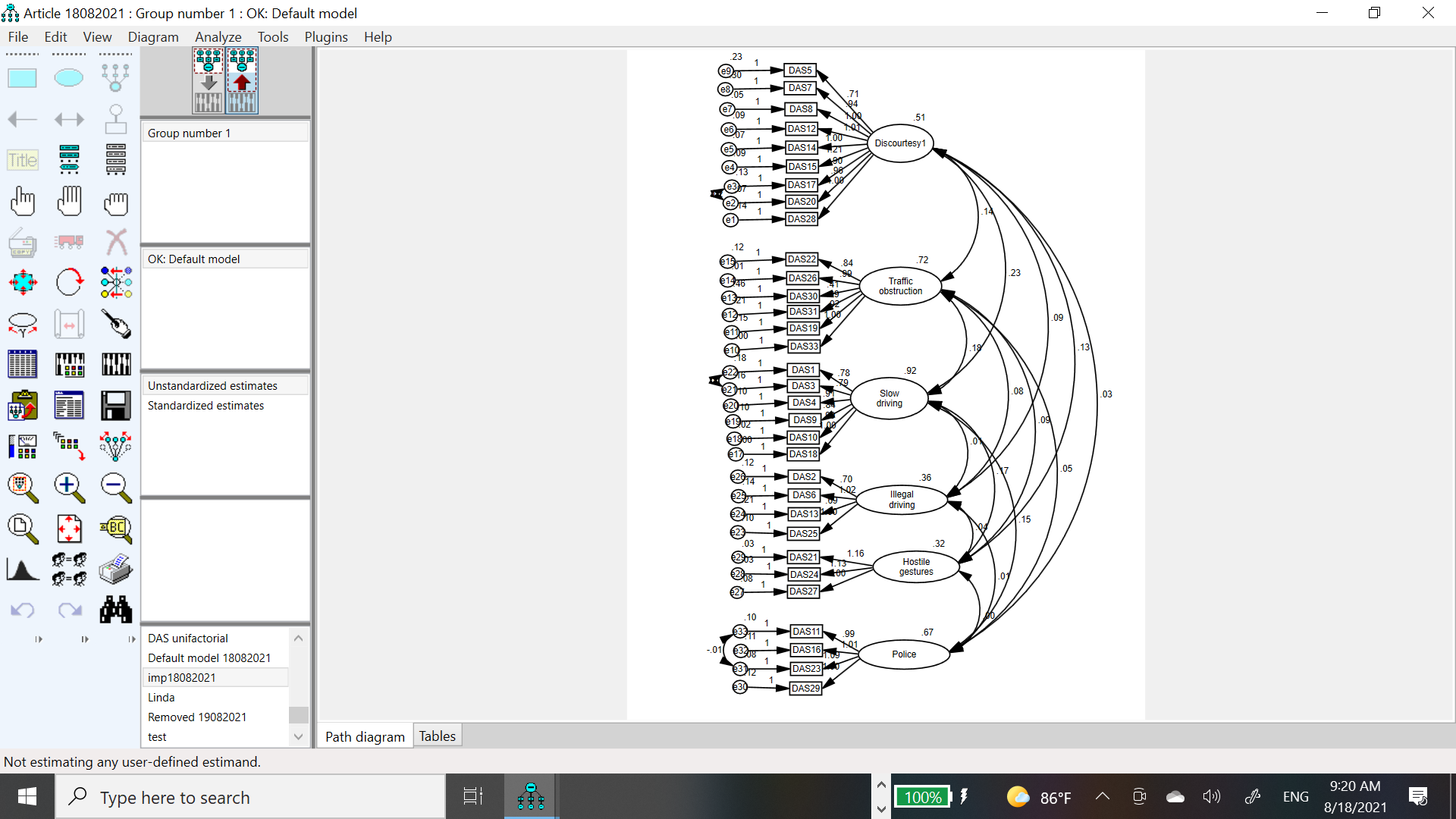


**Annex T1: Correlation matrix for the driver behavior questionnaire (DBQ-L) subscales**

|  | **Hostile gesture** | **Illegal driving** | **Discourtesy** | **Police presence** | **Traffic obstruction** | **Slow driving** | **DAS**  **scale** |
| --- | --- | --- | --- | --- | --- | --- | --- |
| **Hostile gesture** | 1 | 0.287^**^ | 0.341^**^ | 0.010 | 0.217^**^ | 0.309^**^ | 0.478^**^ |
| **Illegal driving** |  | 1 | 0.510^**^ | 0.051 | 0.321^**^ | 0.205^**^ | 0.612^**^ |
| **Discourtesy** |  |  | 1 | 0.064 | 0.274^**^ | 0.396^**^ | 0.775^**^ |
| **Police presence** |  |  |  | 1 | 0.080 | 0.178^**^ | 0.376^**^ |
| **Traffic obstruction** |  |  |  |  | 1 | 0.258^**^ | 0.617^**^ |
| **Slow driving** |  |  |  |  |  | 1 | 0.702^**^ |
| **DAS scale** |  |  |  |  |  |  | 1 |
| **Note:** ** Correlation is significant at the 0.001 level (p<0.01), * Correlation is significant p<0.05 | | | | | | | |

1. Evans, L., *Traffic fatality reductions: United States compared with 25 other countries.* American journal of public health, 2014. **104**(8): p. 1501-1507.

2. Novoa, A.M., et al., *Road safety in the political agenda: the impact on road traffic injuries.* J Epidemiol Community Health, 2011. **65**(3): p. 218-25.

3. Hassen, A., et al., *Risky driving behaviors for road traffic accident among drivers in Mekele city, Northern Ethiopia.* BMC Research Notes, 2011. **4**(1): p. 535.

4. Scott-Parker, B. and O. Oviedo-Trespalacios, *Young driver risky behaviour and predictors of crash risk in Australia, New Zealand and Colombia: Same but different?* Accident Analysis & Prevention, 2017. **99**: p. 30-38.

5. Sümer, N., *Personality and behavioral predictors of traffic accidents: testing a contextual mediated model.* Accid Anal Prev, 2003. **35**(6): p. 949-64.

6. Mizell, L., M. Joint, and D. Connell, *Aggressive driving: Three studies.* 1997.

7. Dahlen, E.R., et al., *Driving anger, sensation seeking, impulsiveness, and boredom proneness in the prediction of unsafe driving.* 2005. **37**(2): p. 341-348.

8. Houston, J.M. and P. Harris, *The Aggressive Driving Behavior Scale: Developing a self-report measure of unsafe driving practices.* 2003.

9. Shinar, D., R.J.A.a. Compton, and prevention, *Aggressive driving: an observational study of driver, vehicle, and situational variables.* 2004. **36**(3): p. 429-437.

10. Useche, S.A., et al., *Trait driving anger and driving styles among Colombian professional drivers.* 2019. **5**(8): p. e02259.

11. Lal, K., *A critical review of the psychophysiology of driver fatigue. Biol Psychol. 2001 Feb;55(3):173-94. doi: 10.1016/s0301-0511(00)00085-5. PMID: 11240213.* 2001, A.

12. May, J.F., C.L.J.T.r.p.F.t.p. Baldwin, and behaviour, *Driver fatigue: The importance of identifying causal factors of fatigue when considering detection and countermeasure technologies.* 2009. **12**(3): p. 218-224.

13. Tao, D., R. Zhang, and X. Qu, *The role of personality traits and driving experience in self-reported risky driving behaviors and accident risk among Chinese drivers.* Accident Analysis & Prevention, 2017. **99**: p. 228-235.

14. Mallia, L., et al., *Crash risk and aberrant driving behaviors among bus drivers: the role of personality and attitudes towards traffic safety.* 2015. **79**: p. 145-151.

15. Nabi, H., et al., *Attitudes associated with behavioral predictors of serious road traffic crashes: results from the GAZEL cohort.* 2007. **13**(1): p. 26-31.

16. Chliaoutakis, J.E., et al., *Aggressive behavior while driving as predictor of self-reported car crashes.* 2002. **33**(4): p. 431-443.

17. Vickers, N.J.J.C.b., *Animal communication: when i’m calling you, will you answer too?* 2017. **27**(14): p. R713-R715.

18. Dahlen, E.R., R.P.J.P. White, and i. differences, *The Big Five factors, sensation seeking, and driving anger in the prediction of unsafe driving.* 2006. **41**(5): p. 903-915.

19. Scherer, K.R., A. Schorr, and T. Johnstone, *Appraisal processes in emotion: Theory, methods, research*. 2001: Oxford University Press.

20. Spielberger, C.D., et al., *Measuring anxiety and anger with the State-Trait Anxiety Inventory (STAI) and the State-Trait Anger Expression Inventory (STAXI)*. 1999: Lawrence Erlbaum Associates Publishers.

21. Deffenbacher, J.L., E.R. Oetting, and R.S.J.P.r. Lynch, *Development of a driving anger scale.* 1994. **74**(1): p. 83-91.

22. Deffenbacher, J., et al., *Driving anger as a psychological construct: Twenty years of research using the Driving Anger Scale.* 2016. **42**: p. 236-247.

23. Zhang, T. and A.H. Chan, *The association between driving anger and driving outcomes: A meta-analysis of evidence from the past twenty years.* Accid Anal Prev, 2016. **90**: p. 50-62.

24. Wickens, C., et al., *Do driver anger and aggression contribute to the odds of a crash? A population-level analysis.* Transportation Research Part F: Traffic Psychology and Behaviour, 2016.

25. Stephens, A.N., J.A.J.C. Groeger, and emotion, *Anger-congruent behaviour transfers across driving situations.* 2011. **25**(8): p. 1423-1438.

26. Feng, Z., et al., *Driving anger and its relationships with type A behavior patterns and trait anger: Differences between professional and non-professional drivers.* PLOS ONE, 2017. **12**: p. e0189793.

27. Lajunen, T., et al., *Dimensions of driver anger, aggressive and highway code violations and their mediation by safety orientation in UK drivers.* 1998. **1**(2): p. 107-121.

28. Sullman, M.J.J.T.R.P.F.T.P. and Behaviour, *Anger amongst New Zealand drivers.* 2006. **9**(3): p. 173-184.

29. Gras, M.E., et al., *Spanish drivers and their aberrant driving behaviours.* 2006. **9**(2): p. 129-137.

30. Villieux, A. and P. Delhomme, *Driving Anger Scale, French adaptation: further evidence of reliability and validity.* Percept Mot Skills, 2007. **104**(3 Pt 1): p. 947-57.

31. Hoggan, B.L. and M.F. Dollard, *Effort-reward imbalance at work and driving anger in an Australian community sample: is there a link between work stress and road rage?* Accid Anal Prev, 2007. **39**(6): p. 1286-95.

32. Yasak, Y. and B. Esiyok, *Anger amongst Turkish drivers: Driving Anger Scale and its adapted, long and short version.* Safety Science, 2009. **47**: p. 138-144.

33. Li, F., et al., *Driving anger in China: Psychometric properties of the Driving Anger Scale (DAS) and its relationship with aggressive driving.* Personality and Individual Differences, 2014. **68**: p. 130-135.

34. McLinton, S.S. and M.F. Dollard, *Work stress and driving anger in Japan.* Accident Analysis & Prevention, 2010. **42**(1): p. 174-181.

35. Przepiorka, A., A. Błachnio, and D. Wiesenthal, *The determinants of driving aggression among Polish drivers.* Transportation Research Part F: Traffic Psychology and Behaviour, 2014. **27**: p. 69–80.

36. Sullman, M.J., A.N. Stephens, and M. Yong, *Driving anger in Malaysia.* Accid Anal Prev, 2014. **71**: p. 1-9.

37. Deffenbacher, J.L., et al., *Driving anger: Correlates and a test of state-trait theory.* 2001. **31**(8): p. 1321-1331.

38. Malta, L.S., et al., *Psychiatric and behavioral problems in aggressive drivers.* 2005. **43**(11): p. 1467-1484.

39. Center, L.T.M., *Road traffic crashes.* 2019.

40. Koller, M., et al., *The process of reconciliation: Evaluation of guidelines for translating quality-of-life questionnaires.* Expert review of pharmacoeconomics & outcomes research, 2012. **12**: p. 189-97.

41. Cuschieri, S., *The STROBE guidelines.* Saudi journal of anaesthesia, 2019. **13**(Suppl 1): p. S31-S34.

42. Comrey, A. and H.J.I. Lee, Publishers, *A first course in factor analysis. Hillsdale, NJ, Lawrence Eribaum Associates.* 1992.

43. Cortina, J.M.J.J.o.a.p., *What is coefficient alpha? An examination of theory and applications.* 1993. **78**(1): p. 98.

44. Yusoff, M.S.B., *ABC of Content Validation and Content Validity Index Calculation.* Education in Medicine Journal, 2019. **11**: p. 49-54.

45. Lawshe, C.H., *A quantitative approach to content validity.* Personnel psychology, 1975. **28**(4): p. 563-575.

46. Ayre, C. and A.J. Scally, *Critical Values for Lawshe’s Content Validity Ratio: Revisiting the Original Methods of Calculation.* Measurement and Evaluation in Counseling and Development, 2013. **47**(1): p. 79-86.

47. Alavi, M., et al., *Chi-square for model fit in confirmatory factor analysis.* 2020.

48. Bentler, P.M.J.P.b., *Comparative fit indexes in structural models.* 1990. **107**(2): p. 238.

49. Byrne, B.M., *Structural equation modeling with EQS and EQS/Windows: Basic concepts, applications, and programming*. 1994: Sage.

50. Barros, L., et al., *Effect size: a statistical basis for clinical practice.* Revista Odonto Ciência, 2018. **33**: p. 84.

51. Brandenburg, S. and M. Oehl, *Driving anger in Germany: Validation of the driving anger scale for German drivers.* Transportation Research Part F: Traffic Psychology and Behaviour, 2021. **81**: p. 317-328.

52. Lajunen, T., D. Parker, and S.G. Stradling, *Dimensions of driver anger, aggressive and highway code violations and their mediation by safety orientation in UK drivers.* Transportation Research Part F: Traffic Psychology and Behaviour, 1998. **1**(2): p. 107-121.

53. Hernández-Hernández, A.M., et al., *Anger while driving in Mexico City.* 2019. **14**(9): p. e0223048.

54. Sullman, M.J.M., et al., *Driving anger in Spain.* Personality and Individual Differences, 2007. **42**(4): p. 701-713.

55. Damjanović, M., et al., *Differences in Driving Anger among Professional Drivers: A Cross-Cultural Study.* International journal of environmental research and public health, 2022. **19**(7): p. 4168.

56. Lawton, R., et al., *The role of affect in predicting social behaviors: The case of road traffic violations.* 1997. **27**(14): p. 1258-1276.

57. Dinh, D., et al., *Examining the roles of multidimensional fatalism on traffic safety attitudes and pedestrian behaviour.* 2020. **124**: p. 104587.

58. Perepjolkina, V. and V.J.S.T. Renge, *Drivers' Age, Gender, Driving Experience, and Aggressiveness as Predictors of Aggressive Driving Behaviour.* 2011. **4**(1): p. 62.
